# Genetically prioritized mitochondrial regulators of advanced renal failure: multi-omic Mendelian randomization and biological plausibility assessment in allograft fibrosis

**DOI:** 10.3389/fimmu.2026.1783844

**Published:** 2026-03-27

**Authors:** Qinghuan Shen, Runmin Ding, Zhiyu Wen, Dengyuan Feng, Jianjian Zhang, Jiawen Liu, Qianguang Han, Li Sun, Hao Chen, Shuang Fei, Zhen Xu, Ruijinlin Hao, Ruoyun Tan

**Affiliations:** 1 The First Affiliated Hospital with Nanjing Medical University, Department of Urology, Nanjing Medical University, Nanjing, China; 2 The Second Affiliated Hospital of Nanjing Medical University, Jiangsu Key Laboratory of Urological Disease Prevention and Treatment, Department of Urology, Nanjing Medical University, Nanjing, China; 3 Nanjing Drum Tower Hospital, Department of Andrology, Nanjing, China; 4 The Affiliated Taizhou People’s Hospital of Nanjing Medical University, Department of Urology, Nanjing Medical University, Taizhou, China; 5 The First Affiliated Hospital with Nanjing Medical University, Department of Anesthesiology, Nanjing Medical University, Nanjing, China

**Keywords:** allograft fibrosis, chronic allograft dysfunction, kidney transplantation, Mendelian randomization, mitochondrial dysfunction, *NDUFA13*

## Abstract

**Background:**

Advanced renal failure remains a major global health burden. Mitochondrial dysfunction is frequently observed during progressive kidney injury and chronic allograft dysfunction (CAD), but observational data cannot distinguish causal involvement from secondary consequences. We applied a multi-omic genetic prioritization framework to evaluate whether inherited variation affecting mitochondrial gene regulation is associated with a proxy phenotype for advanced renal failure and fibrotic allograft remodeling.

**Methods:**

We integrated cis-mQTL (DNA methylation), cis-eQTL (gene expression), and cis-pQTL (plasma protein) data for MitoCarta3.0 genes with a UK Biobank GWAS of kidney transplant recipient status (369 cases, 397,602 controls) as a proxy endpoint for advanced renal failure. Summary-data-based Mendelian randomization (SMR; Wald ratio) was performed using a single lead cis-QTL instrument per gene per layer, with HEIDI heterogeneity testing and Bayesian colocalization to assess whether molecular QTL and outcome signals were consistent with a shared causal variant (PPH4 ≥ 0.70). Because no association survived false discovery rate (FDR) correction across the mitochondrial gene set, we used a tiered, exploratory prioritization scheme based on nominal MR evidence and colocalization. Instrument strength metrics (F-statistics and R²) are reported.

**Results:**

At a nominal threshold (*p* < 0.05; none surviving FDR < 0.05), we observed suggestive SMR associations in the methylation and expression layers, with generally weaker signals in the protein layer. Integrating MR evidence with colocalization support prioritized eight mitochondrial candidate genes (*C20orf72*/*MGME1*, *NDUFA13*, *MRPS18C*, *MTIF3*, *ECHDC1*, *MTHFD1L*, *QDPR*, and *TST*). Translational evaluation showed dysregulation of several prioritized candidates in human CAD allograft tissues and in a murine allogeneic kidney transplantation model of chronic allograft fibrosis. In TGF-β–stimulated HK-2 cells, mitochondrial dysfunction accompanied profibrotic responses, and functional perturbation supported *NDUFA13* as a plausible node linking mitochondrial bioenergetics to fibrotic remodeling.

**Conclusions:**

Given the limited number of outcome cases, the proxy nature of transplant recipient status, and no FDR-significant associations, the genetic results should be interpreted as exploratory and hypothesis-generating rather than causal proof. Nonetheless, multi-omic genetic prioritization with kidney-relevant experimental data highlights mitochondrial pathways as plausible contributors to advanced renal failure and fibrotic allograft remodeling, motivating replication in larger outcome GWAS and kidney-relevant QTL resources.

## 1 Introduction

Despite advances in renal medicine, end-stage renal disease (ESRD) remains a major global health challenge. Kidney transplantation offers optimal therapy for advanced renal failure; however, long-term outcomes remain limited, with approximately 40% of deceased-donor grafts experiencing failure within 10 years post-transplant (1, 2). This underscores the need to clarify pathogenic mechanisms that drive progression to advanced renal failure and chronic allograft dysfunction (3). Ischemia-reperfusion injury (IRI), inherent to the transplantation process, illustrates how acute mitochondrial stress can trigger lasting kidney dysfunction (4–6). Importantly, the severity and persistence of post-transplant injury vary substantially among recipients, suggesting that inherited genetic variants affecting mitochondrial pathways may modulate individual vulnerability to ischemic insults (7). Defining these molecular determinants may help prioritize biological pathways for future mechanistic and translational studies.

Mitochondrial dysfunction represents a central pathophysiological mechanism in kidney disease progression. The kidney’s extraordinary mitochondrial density, among the highest of any organ, reflects its substantial energetic demands and creates a unique vulnerability to bioenergetic perturbations (8–10). During ischemia-reperfusion, mitochondria undergo a pathological cascade from initial ATP depletion to post-reperfusion reactive oxygen species (ROS) generation, culminating in membrane damage, mtDNA release, and activation of cell death pathways (11–15). Damaged mitochondria release mitochondrial damage-associated molecular patterns (mtDAMPs), particularly mtDNA fragments, which activate innate immune receptors and amplify inflammatory responses (16–18). Consistent with these mechanisms, Clinical evidence demonstrates that mitochondrial dysfunction markers, including elevated circulating and urinary mtDNA levels, are strongly associated with adverse kidney outcomes in both acute injury and chronic disease progression to ESRD (19, 20).

Despite these associations, a critical knowledge gap persists regarding the causal relationship between mitochondrial dysfunction and kidney disease progression. Specifically, it remains unknown whether genetic variants affecting mitochondrial pathways directly influence susceptibility to ESRD or whether observed mitochondrial abnormalities merely reflect secondary damage from other primary insults (21). This distinction has profound implications for therapeutic development, as targeting epiphenomena would likely yield limited clinical benefit. Conventional observational studies cannot resolve this uncertainty due to inherent limitations of confounding and reverse causation (22–24). Furthermore, while genetic studies have identified associations with ESRD risk, they have primarily examined immunological pathways, leaving mitochondrial genetic contributions largely unexplored (23).

Mendelian randomization (MR) can strengthen causal inference by using germline genetic variants as instrumental variables for exposures of interest, thereby reducing confounding and reverse causation. However, the strength of inference depends on instrument validity, statistical power, and the suitability of the outcome definition.

We hypothesize that inherited genetic variation affecting key mitochondrial pathways, particularly those involved in energy production, oxidative stress response, and organelle quality control, may influence vulnerability to advanced renal failure and fibrotic allograft remodeling, and that these effects may be mediated through measurable changes in mitochondrial gene regulation.

This investigation sought to prioritize candidate regulators of mitochondrial function for further investigation. Because the UK Biobank outcome used here represents a proxy phenotype and the number of cases is limited, the genetic findings should be interpreted with caution and considered primarily hypothesis-generating. To evaluate their biological relevance in kidney-related settings, we examined prioritized candidates in human CAD allografts, an allogeneic murine renal transplantation model, and TGF-β–stimulated tubular epithelial cells, and performed targeted functional perturbation of a key candidate.

## 2 Results

### 2.1 Initial MR screening of associations with kidney transplant recipient status (proxy for advanced renal failure)

**Figure 1:**
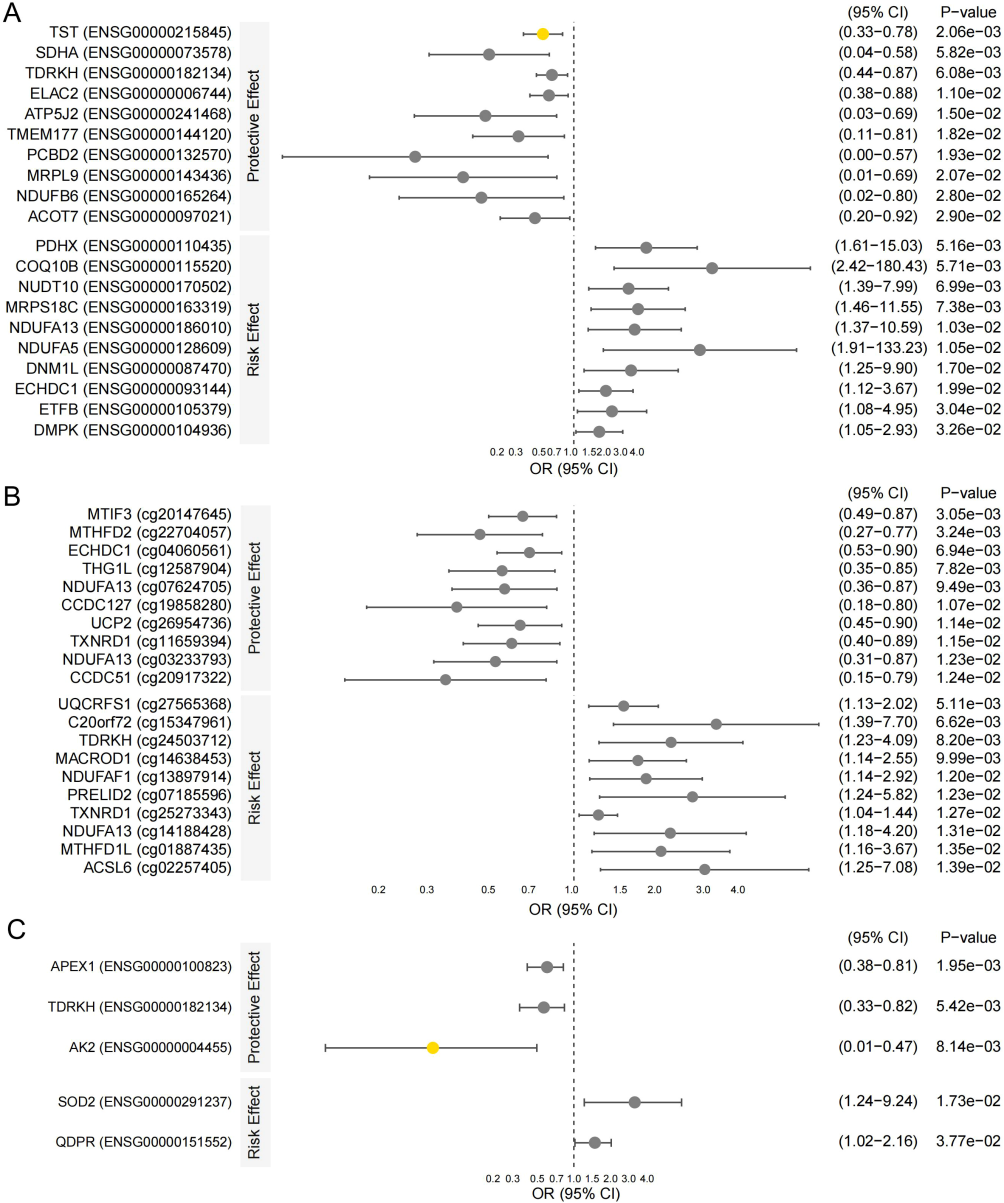
Multi-omic SMR screening across molecular layers. Forest plots summarize nominal SMR associations between mitochondrial gene regulation and kidney transplant recipient status across **(A)** gene expression (eQTL-based SMR), **(B)** DNA methylation (mQTL-based SMR), and **(C)** protein abundance (pQTL-based SMR). Associations are shown for prioritization at a nominal threshold (*p* < 0.05); none survived FDR < 0.05 across the mitochondrial gene set. Colocalization support is indicated by PPH4 ≥ 0.70 (suggestive: 0.50 ≤ PPH4 < 0.70).

Across tested mitochondrial loci, SMR identified nominal associations with kidney transplant recipient status. At a nominal significance threshold of *p* < 0.05 (no locus achieved FDR < 0.05), we detected suggestive associations involving 61 DNA methylation sites and 29 gene transcripts (**Figures 1A, B**). For example, higher genetically predicted expression of *NDUFB6* was associated with a lower odds of transplant recipient status (odds ratio [OR] = 0.13, 95% confidence interval [CI]: 0.02–0.80, *p* = 2.80 × 10^-2^), whereas elevated COQ10B expression was associated with higher odds (OR = 20.9, 95% CI: 2.42–180.43, *p* = 5.71 × 10^-3^) (**Figure 1A**). Given the small number of cases, large point estimates should be interpreted cautiously as they may reflect imprecision and statistical instability rather than large biological effects.

### 2.2 Layer-specific findings in methylation and gene expression

At the DNA methylation level, nominal mQTL–outcome associations were enriched at genes with metabolic functions (**Figure 1B**). Top signals included CpG sites in *MTHFD2* and *TST*, where higher methylation was associated with lower odds of transplant recipient status (e.g., *MTHFD2*, OR = 0.46, 95% CI: 0.27–0.77, *p* = 3.24 × 10^-3^), as well as a site in *C20orf72* where higher methylation was associated with higher odds (OR = 3.27, 95% CI: 1.39–7.70, *p* = 6.62 × 10^-3^). In total, 61 CpG loci showed nominal (*p* < 0.05) associations (**Supplementary Table 1**). At the gene expression level, we identified 29 transcripts with nominal associations (*p* < 0.05), many mapping to mitochondrial or metabolic pathways (**Figure 1A**). For instance, higher expression of *TST* was associated with lower odds (OR = 0.51, 95% CI: 0.33–0.78, *p* = 2.06 × 10^-3^), whereas elevated *PDHX* expression was associated with higher odds (OR = 4.92, 95% CI: 1.61–15.03, *p* = 5.16 × 10^-3^). While several pQTLs showed nominal associations, none survived FDR correction in the initial screen; pQTL results were therefore treated as supportive evidence when available (**Figure 1C**).

### 2.3 Integrative prioritization of candidate genes across omics layers

**Figure 2:**
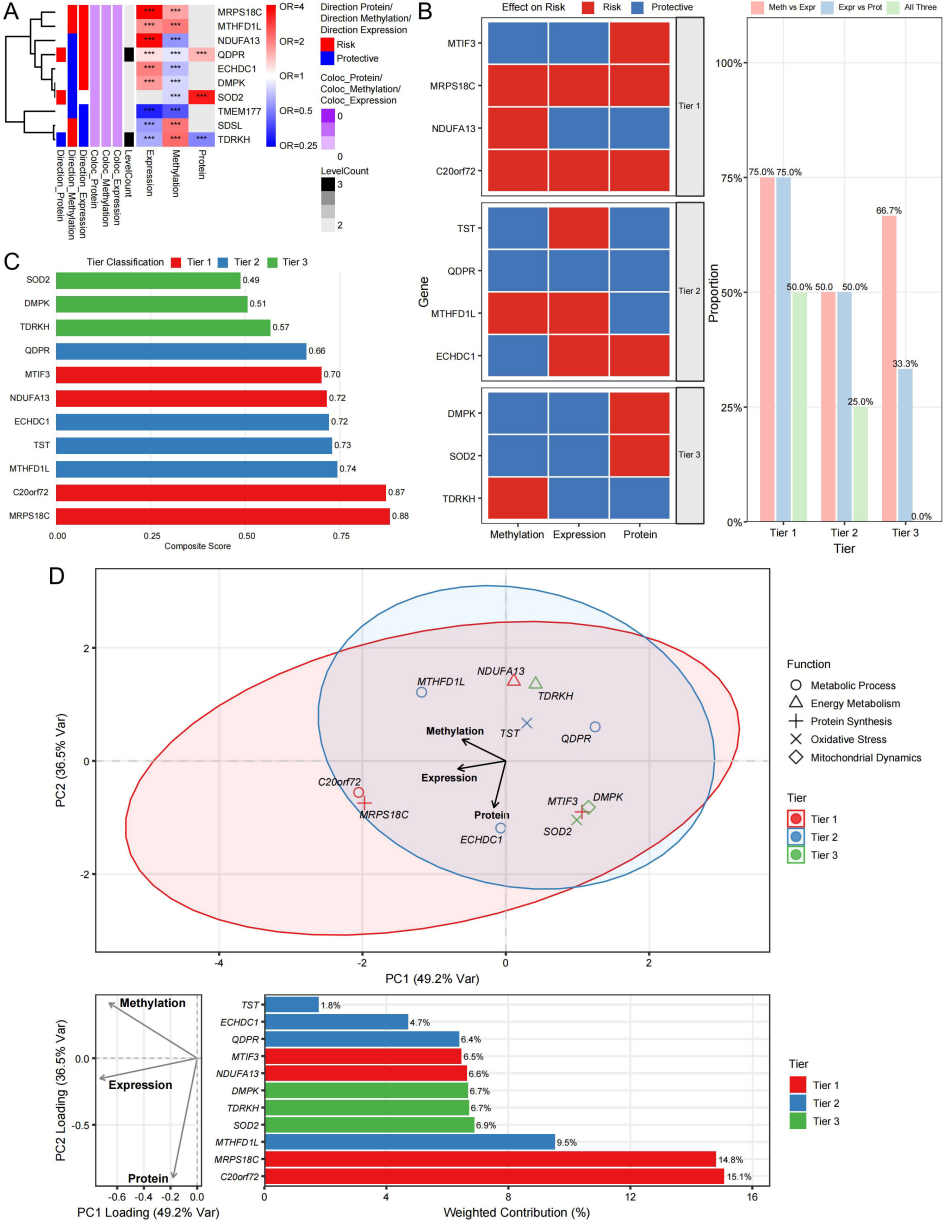
Integrative gene prioritization and tier classification across multi-omic layers. **(A)** Heatmap summarizing evidence across methylation, expression, and protein layers, including effect directions and colocalization support. **(B)** Tier classification of candidates based on nominal SMR evidence, HEIDI filtering, and colocalization support (PPH4). **(C)** Composite prioritization scores integrating multiple evidence dimensions. **(D)** Distribution of composite scores by tier. Tiers are intended for exploratory prioritization because none of the screened associations survived FDR correction. Asterisks denote nominal SMR significance, ***P < 0.001.

**Figure 3:**
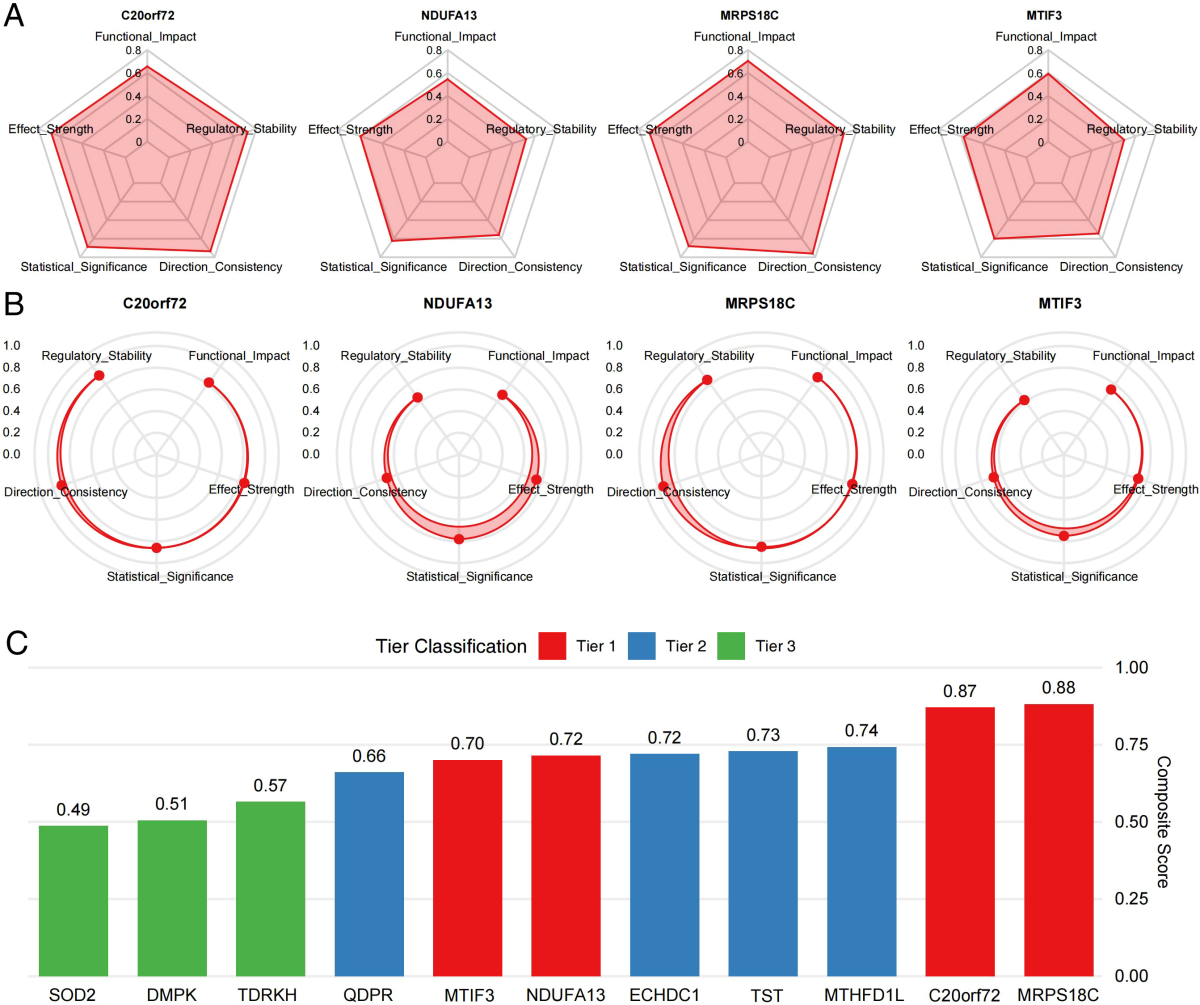
Evidence profiling of prioritized candidates. **(A)** Colocalization support (PPH4) and evidence summaries for prioritized loci. **(B)** Composite prioritization scores ranked by gene. **(C)** Score distributions across tiers. Tiers represent exploratory prioritization rather than confirmatory causal inference.

To refine results, we integrated evidence across methylation, expression, and protein layers using a tiered prioritization framework incorporating nominal SMR evidence and colocalization support (PPH4 ≥ 0.70) (**Figure 2**). This approach prioritized eight Tier 1 mitochondrial candidate genes for follow-up: *C20orf72* (*MGME1*), *NDUFA13*, *MRPS18C*, *MTIF3*, *ECHDC1*, *MTHFD1L*, *QDPR*, and *TST* (**Figure 2A**, **Table 1**). Several candidates showed concordant evidence across methylation and expression layers where instruments were available (**Figure 2B**; **Supplementary Figure 3**). Composite scoring further ranked *MRPS18C, C20orf72/MGME1, MTIF3*, and *NDUFA13* among the highest-priority candidates (**Figures 2C**, **3A–C**). Given the absence of FDR-significant associations across the full mitochondrial gene set, these tiered results should be interpreted exclusively as exploratory prioritization of candidate genes, rather than confirmatory causal evidence.

**Table 1:** Tier 1 mitochondrial candidate genes prioritized by multi-omic SMR and colocalization support (exploratory).

Gene	Putative mitochondrial process	Evidence layers	Lead SNP (eQTL)	Lead SNP (mQTL)	Lead SNP (pQTL)	Colocalization	Tier
C20orf72 (MGME1)	mtDNA maintenance	mQTL		rs118056927 (20:33107241)		PPH4 ≥ 0.70 in ≥1 layer	1
NDUFA13	Complex I/electron transport	eQTL, mQTL	rs11085264 (19:19621780)	rs11085264 (19:19621780)		PPH4 ≥ 0.70 in ≥1 layer	1
MRPS18C	Mitochondrial translation	eQTL, mQTL	rs1565909 (4:84400330)	rs1565909 (4:84400330)		PPH4 ≥ 0.70 in ≥1 layer	1
MTIF3	Mitochondrial translation initiation	mQTL		rs9512686 (13:27992461)		PPH4 ≥ 0.70 in ≥1 layer	1
ECHDC1	Mitochondrial metabolism	eQTL, mQTL	rs9388571 (6:127706262)	rs9388571 (6:127706262)		PPH4 ≥ 0.70 in ≥1 layer	1
MTHFD1L	One-carbon metabolism	eQTL, mQTL	rs13201018 (6:151211007)	rs13201018 (6:151211007)		PPH4 ≥ 0.70 in ≥1 layer	1
QDPR	Cofactor regeneration/redox	eQTL, mQTL, pQTL	rs10020773 (4:17526682)	rs7661303 (4:17518920)	rs7661303 (4:17518920)	PPH4 ≥ 0.70 in ≥1 layer	1
TST	Sulfur metabolism/detoxification	eQTL	rs7290003 (22:37408829)			PPH4 ≥ 0.70 in ≥1 layer	1

### 2.4 Functional enrichment and mechanistic insights

**Figure 4:**
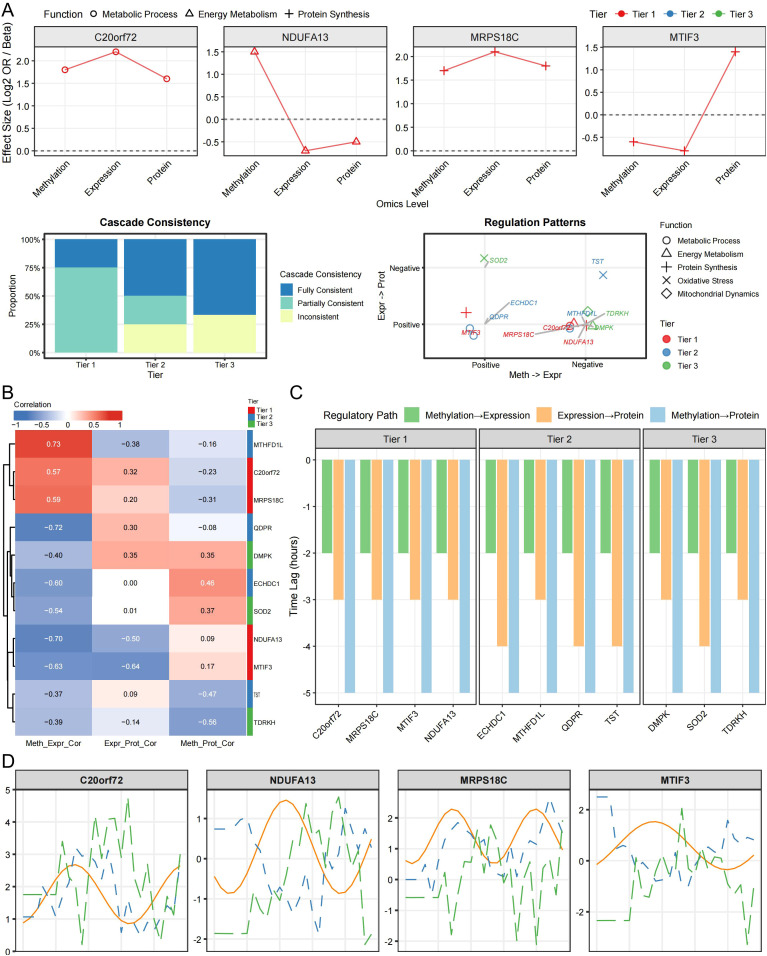
Cross-layer patterns among prioritized candidates. **(A)** Effect estimates across methylation, expression, and protein layers. **(B)** Correlation of effect estimates between layers. **(C)** Exploratory comparison of cross-layer ordering metrics (descriptive; not a direct measurement of temporal lag). **(D)** Cross-layer consistency classifications for prioritized candidates. Associations are shown for prioritization at nominal *p* < 0.05; none survived FDR < 0.05 across the mitochondrial gene set. Colocalization support is indicated by PPH4 ≥ 0.70 (suggestive: 0.50 ≤ PPH4 < 0.70).

**Figure 5:**
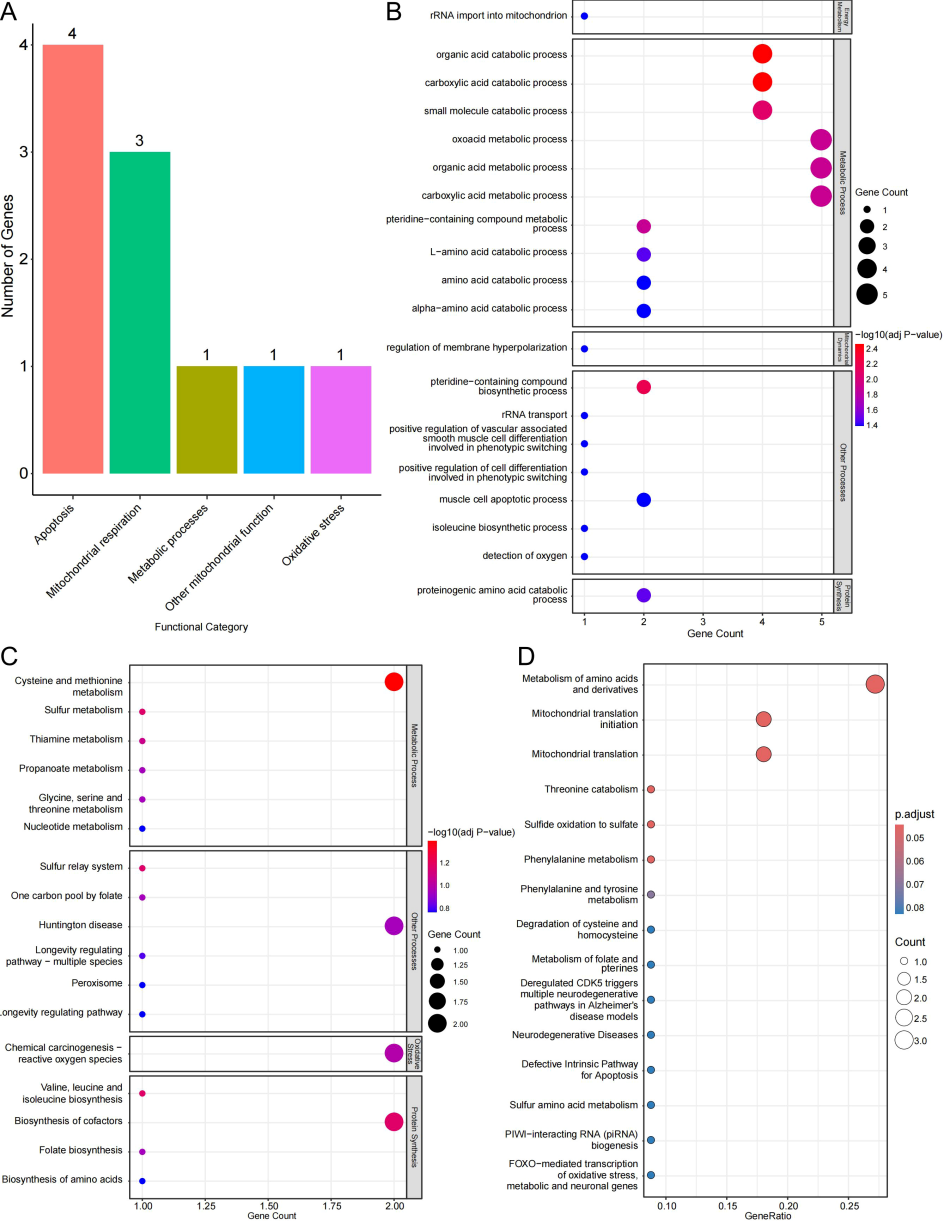
Functional enrichment analysis of prioritized mitochondrial candidate genes. **(A)** Distribution of prioritized genes across major functional categories. **(B)** GO biological process enrichment analysis showing over-representation of mitochondrial and metabolic processes. **(C)** KEGG pathway enrichment analysis highlighting sulfur amino acid metabolism, cofactor biosynthesis, reactive oxygen species–related pathways, and longevity-associated pathways. **(D)** Reactome pathway enrichment analysis indicating enrichment in amino acid metabolism, mitochondrial translation, apoptosis-related pathways, and oxidative stress–associated transcriptional regulation. Bubble size indicates gene count; bubble color indicates enrichment significance.

Pathway enrichment analysis of prioritized candidates revealed convergence on key mitochondrial and metabolic processes (**Figures 4A**, **5**). Several Tier 1 candidates encode proteins involved in intramitochondrial protein synthesis, such as *MRPS18C*, a mitochondrial ribosomal subunit, and *MTIF3*, a translation initiation factor, as well as electron transport components, including *NDUFA13*, a Complex I subunit (**Figure 5B**). Other genes contribute to one-carbon metabolism (*MTHFD1L*) or mediate cellular detoxification and redox balance (e.g., TST in sulfur metabolism, *ECHDC1* in fatty acid metabolism, and *QDPR* in cofactor regeneration), consistent with the functional categories enriched among prioritized genes (**Figures 5C, D**).

Although identified through single-instrument SMR at distinct loci, prioritized candidates collectively mapped to a mechanistic network centered on mitochondrial energy metabolism, metabolic homeostasis, and cellular stress responses (**Figures 2A, B**). Cross-layer effect estimates were directionally coherent for several candidates and are summarized in **Figure 4B–D** and **Supplementary Figure 3**. The cross-omic patterns should be interpreted descriptively and as prioritization signals rather than as direct evidence of temporal ordering or definitive molecular cascades.

### 2.5 Supportive checks and transparency for genetic prioritization

Given the single-instrument SMR design, we used the HEIDI heterogeneity test and Bayesian colocalization as complementary safeguards against linkage-driven associations and distinct causal variants. All Tier 1 candidates showed colocalization support (PPH4 ≥ 0.70) in at least one molecular layer (**Figure 3A**). To address concerns about weak-instrument bias, we report instrument strength metrics (F-statistics and R²) for all lead cis-QTL instruments used in SMR for prioritized genes (**Supplementary Table 4**). Importantly, in exploratory sensitivity analyses utilizing more stringent thresholds, core candidates such as *MRPS18C* (eQTL PPH4 = 0.82) and *C20orf72* (eQTL PPH4 = 0.81) maintained robust colocalization support (PPH4 > 0.80), reinforcing their prioritization. Nonetheless, HEIDI/colocalization do not fully exclude residual horizontal pleiotropy or winner’s curse, and the limited number of outcome cases can yield imprecise estimates; therefore, the genetic results are interpreted as exploratory prioritization. These genetic results motivated downstream evaluation of prioritized candidates in kidney-relevant experimental settings. The following sections assess whether candidates show dysregulation in fibrotic allografts and whether perturbation of a key candidate influences profibrotic remodeling and mitochondrial function.

### 2.6 Translational evaluation of prioritized mitochondrial genes in human CAD allografts and a murine allogeneic transplantation model

**Figure 6:**
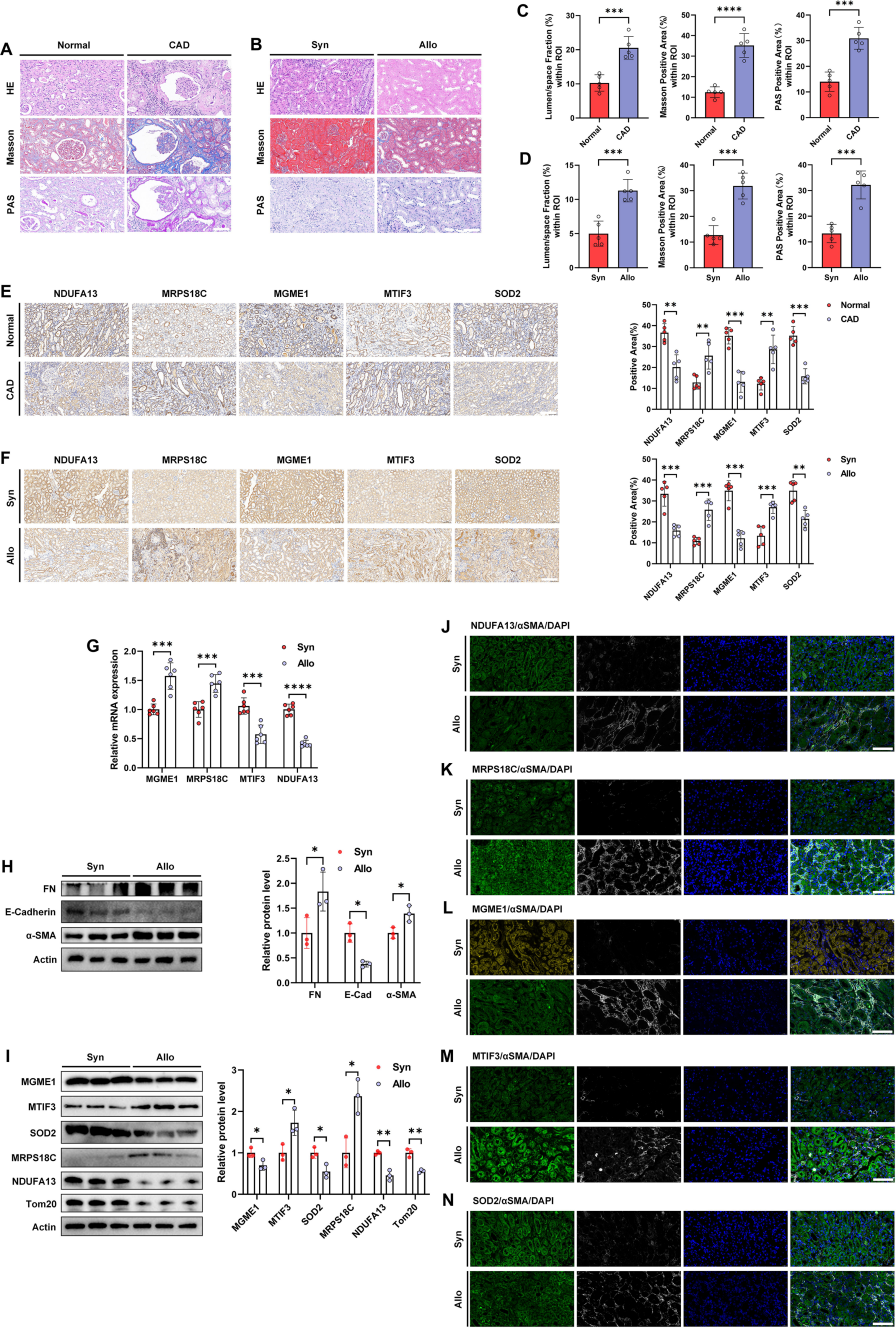
Selected mitochondrial candidates show dysregulation in CAD and allogeneic allografts. **(A)** Representative images of HE, Masson, and PAS staining in human kidney tissues from the Normal and CAD groups (n = 5; bar = 100 μm). **(B)** Representative images of HE, Masson trichrome, and PAS staining in transplanted kidney tissues from the syngeneic (Syn) and allogeneic (Allo) mouse groups (n = 5; bar = 100 μm). **(C)** Statistical graphs of semi-quantitative analyses of luminal space fraction and Masson and PAS positive areas in human kidney tissues from the Normal and CAD groups (n = 5). **(D)** Statistical graphs of semi-quantitative analyses of luminal space fraction and Masson- and PAS-positive areas in transplanted kidney tissues from the Syn and Allo mouse groups (n = 5). **(E)** Representative IHC images and statistical graphs of semi-quantitative analyses of NDUFA13, MRPS18C, MGME1, MTIF3, and SOD2 expression in human kidney tissues from the Normal and CAD groups (n = 5; bar = 200 μm). **(F)** Representative IHC images and statistical graphs of semi-quantitative analyses of NDUFA13, MRPS18C, MGME1, MTIF3, and SOD2 expression in transplanted kidney tissues from the Syn and Allo mouse groups (n = 5; bar = 200 μm). **(G)** RT–qPCR analysis of *MGME1*, *MRPS18C*, *MTIF3*, and *NDUFA13* mRNA expression in transplanted kidney tissues from the Syn and Allo mouse groups (n = 6). **(H)** The results of western blot analyses and quantitative analyses of the relative abundance of fibrotic/EMT-related markers (fibronectin, E-cadherin, and α-SMA) in transplanted kidney tissues from the Syn (n = 3) and Allo (n = 3) groups. **(I)** The results of western blot analyses and quantitative analyses of the relative abundance of MGME1, MTIF3, SOD2, MRPS18C, NDUFA13 and Tom20 expression in transplanted kidney tissues from the Syn (n = 3) and Allo (n = 3) groups. **(J–N)** Representative images showing colocalization of α-SMA with NDUFA13 **(J)**, MRPS18C **(K)**, MGME1 **(L)**, MTIF3 **(M)**, and SOD2 **(N)** in transplanted kidney tissues from the Syn and Allo mouse groups by IF staining; nuclei were counterstained with DAPI (n = 5; bar = 80 μm). *P < 0.05, **P < 0.01, ***P < 0.001, ****P < 0.0001.

To assess translational relevance, we examined whether prioritized mitochondrial candidates show consistent dysregulation in fibrotic kidney allografts from CAD patients and in a chronic renal allograft interstitial fibrosis mouse model. BALB/C mice were used as recipients and transplanted with kidneys from BALB/C (syngeneic, Syn) or C57BL/6 (allogeneic, Allo) donors. Representative HE, Masson, and PAS staining revealed pronounced histopathological injury and interstitial fibrosis in human CAD allograft tissues (**Figures 6A, C**). Compared with the Syn group, Allo allografts demonstrated interstitial inflammatory cell infiltration, glomerulosclerosis, and pronounced interstitial fibrosis on HE, Masson, and PAS staining (**Figures 6B, D**).

We next evaluated a subset of candidates at the protein level, focusing on Tier 1 candidates where antibodies were available (NDUFA13, MGME1, MRPS18C, and MTIF3) and including SOD2 as a mitochondrial oxidative stress marker. Immunohistochemistry (IHC) staining revealed decreased expressions of NDUFA13, MGME1, and SOD2, together with increased expressions of MRPS18C and MTIF3 in the CAD and Allo groups (**Figures 6E, F**). At the transcript level, *MGME1* and *MRPS18C* mRNA levels were increased, whereas *MTIF3* and *NDUFA13* mRNA levels were decreased in the Allo group (**Figure 6G**), indicating potential post-transcriptional regulation for some candidates. Moreover, western blotting assays showed increased expressions of fibronectin and α-SMA, along with decreased E-cadherin expression in the Allo group (**Figure 6H**). These patterns were corroborated by western blotting of mitochondrial proteins, which showed decreased expressions of NDUFA13, MGME1, SOD2, and Tom20, along with increased expressions of MRPS18C and MTIF3 in the Allo group (**Figure 6I**). Finally, IF co-staining with α-SMA supported consistent directional changes for these selected proteins in the Allo group (**Figures 6J–N**).

### 2.7 Evaluation of prioritized mitochondrial genes and mitochondrial dysfunction in HK-2 cells

**Figure 7:**
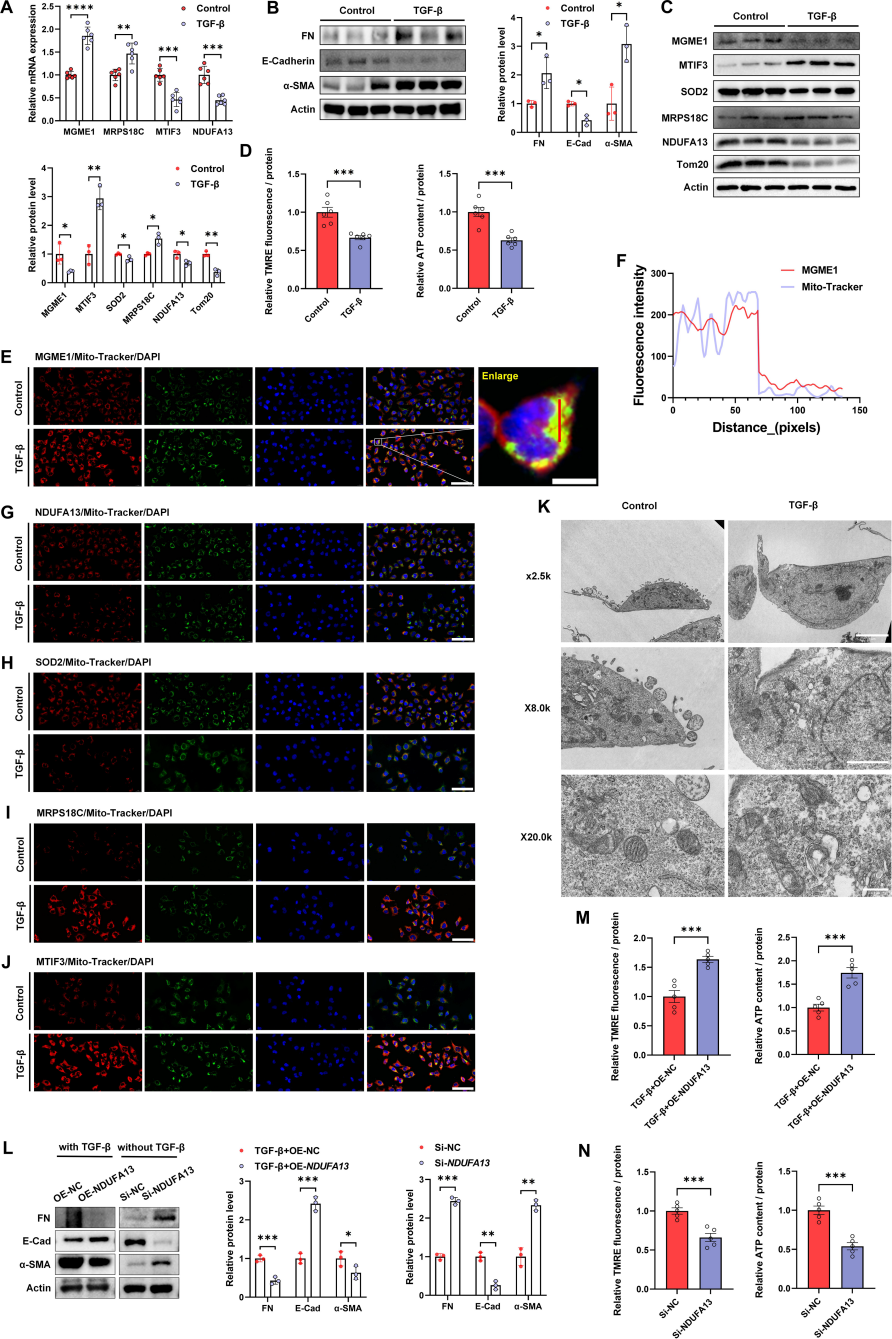
NDUFA13 modulates mitochondrial dysfunction and profibrotic responses in HK-2 cells. **(A)** RT–qPCR analysis of *MGME1*, *MRPS18C*, *MTIF3*, and *NDUFA13* mRNA expression in HK-2 cells treated with TGF-β or vehicle control (n = 6). **(B)** The results of western blot analyses and quantitative analyses of the relative abundance of fibrotic/EMT-related markers (fibronectin, E-cadherin, and α-SMA) in HK-2 cells treated with TGF-β or vehicle control (n = 3). **(C)** The results of western blot analyses of mitochondrial candidate proteins (MGME1, MTIF3, SOD2, MRPS18C, NDUFA13, and Tom20) in HK-2 cells treated with TGF-β or vehicle control (n = 3). **(D)** Quantitative analyses of the relative TMRE fluorescence intensity and the relative cellular ATP content in HK-2 cells treated with TGF-β or vehicle control (n = 6). **(E)** Representative IF images showing colocalization of MGME1 with MitoTracker in HK-2 cells treated with TGF-β or vehicle control; nuclei were counterstained with DAPI (n = 5; bar = 80 μm). The enlarged view is shown on the right (bar = 10 μm). **(F)** Line-scan analysis of fluorescence intensity profiles for MGME1 and MitoTracker signals along the indicated line in HK-2 cells (representative of n = 5). **(G–J)** Representative immunofluorescence images showing colocalization of NDUFA13 **(G)**, SOD2 **(H)**, MRPS18C **(I)**, and MTIF3 **(J)** with MitoTracker in HK-2 cells treated with TGF-β or vehicle control; nuclei were counterstained with DAPI (n = 5; bar = 80 μm). **(K)** Representative transmission electron microscopy images showing mitochondrial ultrastructure in HK-2 cells treated with TGF-β or vehicle control (n = 3). **(L)** The results of western blot analyses and quantitative analyses of the relative abundance of fibrotic/EMT-related markers in HK-2 cells transfected with NDUFA13 overexpression plasmid or control vector under TGF-β stimulation, or cultured without TGF-β as indicated (n = 3). **(M)** Quantitative analyses of the relative TMRE fluorescence intensity and relative ATP content in HK-2 cells transfected with NDUFA13 overexpression plasmid or control vector under TGF-β stimulation (n = 6). **(N)** Quantitative analyses of the relative TMRE fluorescence intensity and relative ATP content in HK-2 cells transfected with NDUFA13 siRNA or negative control siRNA under TGF-β stimulation (n = 6). *P < 0.05, **P < 0.01, ***P < 0.001, ****P < 0.0001.

To further evaluate prioritized mitochondrial genes at the cellular level, HK-2 cells were stimulated with TGF-β to induce a profibrotic phenotype. TGF-β treatment markedly altered the mRNA expression of prioritized mitochondrial genes, with increased *MGME1* and *MRPS18C* expression and decreased *MTIF3* and *NDUFA13* expression (**Figure 7A**). Moreover, western blotting assays showed increased expressions of fibronectin and α-SMA, along with decreased E-cadherin expression in the TGF-β group (**Figure 7B**). In parallel, western blotting analyses demonstrated alterations in mitochondrial regulatory proteins (MGME1, MTIF3, SOD2, MRPS18C, and NDUFA13) and the mitochondrial outer membrane marker Tom20 (**Figure 7C**), suggesting impaired mitochondrial integrity under profibrotic conditions. Functionally, TGF-β stimulation resulted in loss of mitochondrial membrane potential (TMRE) and reduced intracellular ATP levels, indicating impaired mitochondrial bioenergetic capacity (**Figure 7D**). We evaluated the mitochondrial colocalization of prioritized candidate proteins via immunofluorescence staining and fluorescence co-localization analysis (**Figures 7E–J**). We assessed the ultrastructural alterations of mitochondria under TGF-β stimulation via transmission electron microscopy (**Figure 7K**). We further tested a mechanistically plausible candidate (NDUFA13): knockdown promoted profibrotic marker changes under basal conditions, while overexpression attenuated TGF-β–induced profibrotic responses and partially restored mitochondrial function (**Figures 7L–N**).

To further characterize the mitochondrial-specific function of *NDUFA13* in renal fibrogenesis, we assessed mitochondrial superoxide levels in HK-2 cells using the mitochondria-targeted probe MitoSOX Red. TGF-β stimulation induced a marked increase in mitochondrial superoxide production in control vector-transfected cells, which was significantly blunted by *NDUFA13* overexpression. Notably, *NDUFA13* silencing alone was sufficient to drive a prominent elevation in mitochondrial superoxide levels, even in the absence of TGF-β stimulation (**Supplementary Figure 7**). These findings, together with our prior observations of impaired mitochondrial membrane potential and reduced ATP synthesis in TGF-β-treated cells, demonstrate that *NDUFA13* preserves mitochondrial homeostasis by suppressing mitochondrial oxidative stress, thereby attenuating profibrotic responses in tubular epithelial cells.

## 3 Discussion

In this multi-omic SMR study, we integrated blood-derived cis-mQTL, cis-eQTL, and cis-pQTL resources with a UK Biobank GWAS of kidney transplant recipient status to prioritize mitochondrial regulatory candidates associated with a proxy phenotype for advanced renal failure treated with transplantation (25–27). Using a tiered framework incorporating nominal SMR evidence and colocalization support, we prioritized eight mitochondrial candidates (*C20orf72*/*MGME1*, *NDUFA13*, *MRPS18C*, *MTIF3*, *ECHDC1*, *MTHFD1L*, *QDPR*, and *TST*) for downstream evaluation.

These candidates mapped to biologically plausible mitochondrial processes, including mitochondrial protein synthesis (*MRPS18C*, *MTIF3*), electron transport chain function (*NDUFA13*), mtDNA maintenance (*C20orf72*/*MGME1*), and metabolic/redox pathways (*MTHFD1L*, *TST*, *ECHDC1*, *QDPR*) *(*28–30). The convergence on core mitochondrial functions is consistent with the kidney’s high energy demand and the known involvement of mitochondrial dysfunction in ischemia-reperfusion injury and chronic allograft fibrosis (31).

Translational experiments provide supportive biological plausibility in kidney-related contexts. Several candidates exhibited dysregulated expression in human CAD allografts and in chronically rejected murine allografts. Notably, we observed partial discordance between transcript and protein levels for certain candidates (e.g., *MGME1* and *MRPS18C*) in fibrotic tissues. This likely reflects a compensatory transcriptional response to severe ‘mitochondrial translation stress’ during chronic injury, wherein impaired assembly, mistranslation, or accelerated degradation of mitochondrial complexes triggers post-transcriptional uncoupling. In HK-2 cells, TGF-β–induced profibrotic remodeling was accompanied by mitochondrial dysfunction. Targeted perturbation supported *NDUFA13* as a functional node linking mitochondrial bioenergetics to profibrotic marker expression. These experiments do not validate a genetic causal pathway per se, but they strengthen mechanistic plausibility for prioritized candidates in relevant tissue contexts.

The genetic component has important limitations that constrain causal interpretation. First, the outcome GWAS included only 369 cases and used kidney transplant recipient status as a proxy phenotype; this endpoint is influenced by transplant eligibility, access to care, survivorship, and underlying disease heterogeneity, introducing potential selection/collider bias. Furthermore, patients on dialysis who never underwent transplantation might be categorized as controls within the biobank population, potentially introducing misclassification bias. Second, no association survived FDR correction across the mitochondrial gene set, so genetic results should be interpreted as exploratory and hypothesis-generating. Third, SMR relied on a single lead cis-QTL instrument per gene per layer; multi-instrument pleiotropy-robust MR methods are not applicable, and HEIDI/colocalization do not fully exclude residual horizontal pleiotropy or winner’s curse (32). To improve transparency, we report instrument strength metrics (F-statistics and R²) for lead instruments (**Supplementary Table 4**). Fourth, exposures were derived from peripheral blood QTL resources, which may not reflect kidney- or cell-type-specific regulatory architecture under disease conditions (33). Finally, pQTL instruments from UKB−PPP may partially overlap with UK Biobank outcome samples, and the CAD tissue cohort is small without adjustment for clinical covariates or independent replication. Furthermore, while our *in vitro* HK-2 model provides mechanistic insights into tubular epithelial profibrotic remodeling, it is a simplified system that inherently lacks the complex allo-immune and microvascular elements driving chronic allograft dysfunction *in vivo (*34).

In summary, our results nominate mitochondrial pathways and specific candidate genes for future work in larger outcome GWAS, kidney-relevant QTL resources, and mechanistic studies designed to test kidney-specific mediation. The present study should be viewed as a multi-omic genetic prioritization effort that generates testable hypotheses linking mitochondrial regulation to advanced renal failure and fibrotic allograft remodeling.

## 4 Methods

### 4.1 Study design and rationale

**Figure 8:**
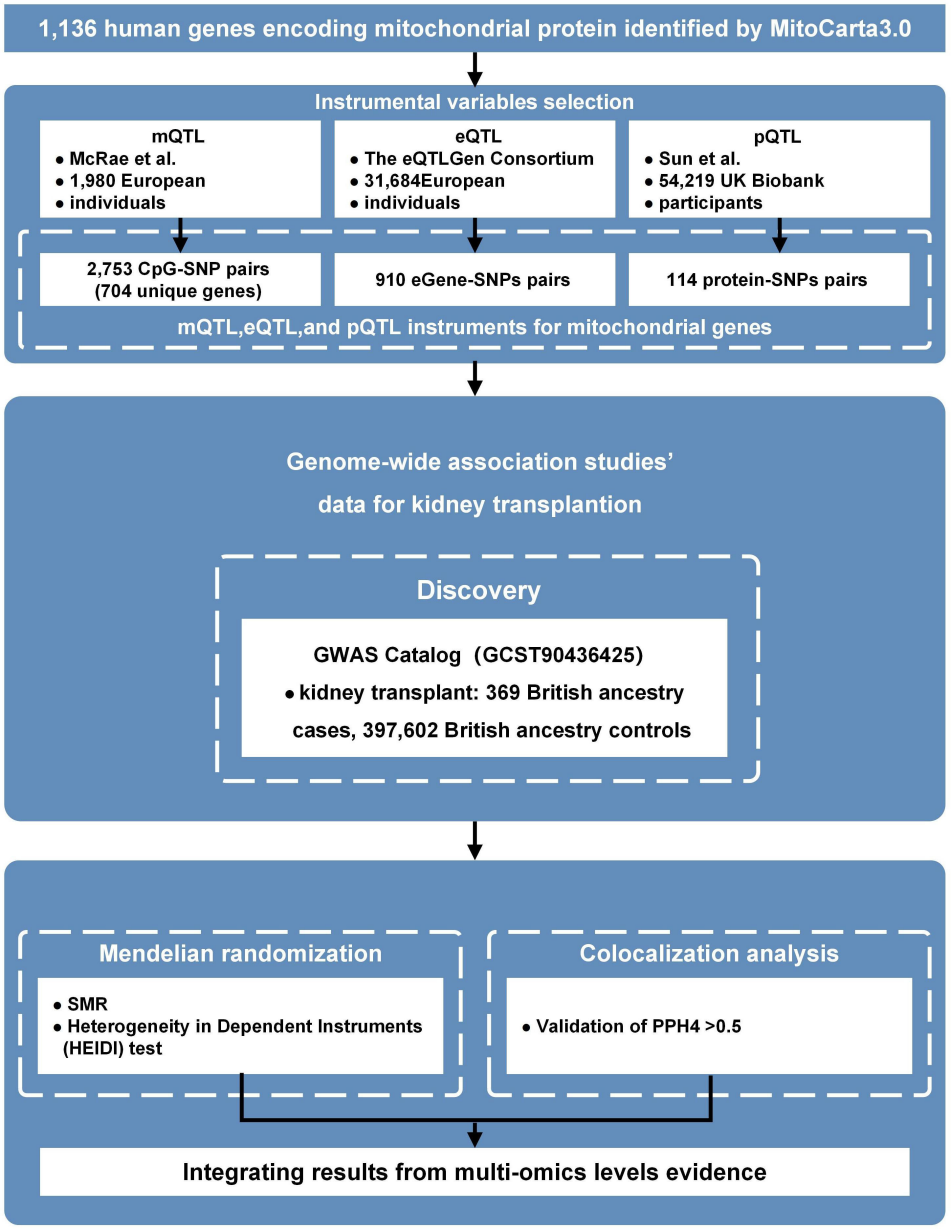
Study design of the multi-omic Mendelian randomization analysis. The workflow begins with 1,136 mitochondrial protein-coding genes from the MitoCarta3.0 database. Lead cis-QTL instruments were selected from multi-omic resources: mQTL CpG–SNP pairs (blood DNA methylation QTLs from McRae et al.), eQTL gene–SNP pairs (whole-blood expression QTLs from the eQTLGen Consortium), and pQTL protein–SNP pairs (UKB−PPP plasma protein QTLs from Sun et al.). Candidate instruments were tested for association with kidney transplant recipient status in UK Biobank (369 cases and 397,602 controls of European ancestry) using summary-data-based Mendelian randomization (SMR) with HEIDI as a supportive heterogeneity check. Bayesian colocalization was used to assess whether molecular and outcome signals were consistent with a shared causal variant. Evidence across layers was integrated to prioritize candidate mitochondrial regulators for downstream evaluation. (SMR, summary-based Mendelian randomization; QTL, quantitative trait locus; SNP, single nucleotide polymorphism; GWAS, genome-wide association study; PPH4, posterior probability of a shared causal variant).

**Figure 8** shows the overall study design. We used kidney transplant recipient status in UK Biobank as a proxy phenotype for advanced renal failure treated with transplantation. We conducted a multi-omic two-sample MR/SMR analysis to prioritize mitochondrial gene regulatory features whose genetically predicted variation was associated with this proxy outcome (35, 36). We integrated three layers of molecular QTL data, DNA methylation (mQTL), gene expression (eQTL), and plasma protein abundance (pQTL), restricted to MitoCarta3.0 genes (37). Bayesian colocalization was used to assess whether molecular QTL and outcome associations were consistent with a shared causal variant. Because the outcome is a proxy phenotype and case counts are limited, results are interpreted as exploratory prioritization.

A subset of individuals may receive pre-emptive transplantation; therefore, transplant recipient status cannot fully distinguish ESRD incidence from late-stage chronic kidney disease treated pre-emptively. This phenotype definition and potential selection effects are considered key limitations.

### 4.2 Mitochondrial gene definition

Mitochondrial genes were defined according to the MitoCarta3.0 reference catalog, which lists 1,136 human genes encoding proteins with strong mitochondrial localization evidence (38). We used this gene set to filter features in the QTL resources. Specifically, we identified all available DNA methylation probes, gene transcripts, and plasma proteins in the QTL datasets that mapped to MitoCarta3.0 genes. Only these mitochondrial-related QTL associations were carried forward into the MR and colocalization analyses.

### 4.3 Outcome GWAS data

The outcome of interest was kidney transplant recipient status—a binary trait indicating whether an individual had undergone kidney transplantation. We used this endpoint as a proxy for advanced renal failure treated with transplantation. Status was determined in UK Biobank using a combination of hospital episode statistics (ICD-10 code Z94.0), self-reported transplantation status, and registry linkage (39). Summary association statistics were obtained from a UK Biobank GWAS (369 cases, 397,602 controls of European ancestry), adjusted for age, sex, genotyping array, and genetic principal components. We used the reported GWAS summary effect sizes (beta coefficients and standard errors) in SMR analyses (40).

### 4.4 Molecular QTL data sources

We retrieved publicly available cis-QTL summary statistics to serve as exposure data for the SMR analysis, including whole-blood DNA methylation QTLs (mQTL; McRae et al. (41); N = 1,980), whole-blood gene expression QTLs from the eQTLGen consortium (eQTL; N = 31,684) (42), and plasma protein QTLs from the UK Biobank Pharma Proteomics Project (UKB−PPP; Sun et al. (43); N = 54,219). All datasets were based primarily on individuals of European ancestry. Each QTL dataset included SNP–trait associations within ±1 Mb of the gene (cis-acting), and was filtered to retain only features corresponding to MitoCarta3.0 genes. When multiple probes or transcripts mapped to the same gene, each was analyzed separately. We note that partial sample overlap is possible between UKB−PPP pQTL and the UK Biobank outcome GWAS, and pQTL findings are interpreted cautiously as supportive evidence.

### 4.5 Mendelian randomization analysis

We used summary-level SMR to test whether genetically predicted variation in each mitochondrial gene’s methylation, expression, or protein abundance was associated with kidney transplant recipient status. For each gene in each QTL dataset, we selected the top cis-QTL SNP as the instrumental variant (lowest p-value within ±1 Mb, genome-wide significant at *p* < 5 × 10^-8^), and included at most one instrument per gene per omics layer. SMR effect estimates were computed using a Wald ratio framework implemented in GCTA/SMR (v1.3.1) (44–46). We applied Benjamini–Hochberg FDR control (α = 0.05) across all tested mitochondrial features for screening; because no association survived FDR correction, subsequent tiering is presented as exploratory prioritization based on nominal evidence.

We used the HEIDI heterogeneity test as a supportive check to distinguish a shared-variant model from linkage (P-HEIDI ≥ 0.01). We also quantified instrument strength for each lead cis-QTL using the F-statistic (F = (β_QTL/SE_QTL)²) and estimated variance explained (R² = F/(F + N − 2)), where N denotes the effective QTL sample size for the specific SNP–feature association. F-statistics and R² values are reported in **Supplementary Table 4** to facilitate assessment of weak-instrument concerns. As in all single-instrument designs, residual horizontal pleiotropy and winner’s curse cannot be fully excluded, particularly given limited outcome GWAS power.

### 4.6 Colocalization analysis

For loci prioritized for follow-up (nominal SMR *p* < 0.05 and passing HEIDI), we performed colocalization analysis to test whether the molecular QTL and outcome associations were consistent with a shared causal variant. We used the R package coloc to compute the posterior probability of a shared causal variant (PPH4) using all SNPs in the cis-region (± 1 Mb for eQTL/pQTL; ± 0.5 Mb for mQTL) present in both datasets (47–49). Colocalization support was defined as PPH4 ≥ 0.70 (suggestive: 0.50 ≤ PPH4 < 0.70).

### 4.7 Multi-omic gene prioritization

To prioritize candidates for downstream biological evaluation, we integrated nominal SMR evidence, HEIDI results, and colocalization across omics layers and classified genes into evidence tiers (50). Because no association survived FDR correction, these tiers are intended for exploratory prioritization rather than confirmatory causal inference.

- Tier 1: Genes with nominal SMR evidence (*p* < 0.05) and colocalization support (PPH4 ≥ 0.70) in at least one omics layer (mQTL/eQTL/pQTL); multi-layer concordance was noted when available (51).
- Tier 2: Genes with nominal SMR evidence (*p* < 0.05) and suggestive colocalization (0.50 ≤ PPH4 < 0.70) (52).
- Tier 3: Genes with nominal SMR evidence (*p* < 0.05) but without colocalization support, or with incomplete supporting evidence.

Genes below Tier 3 were not pursued further. This framework yields a concise list of prioritized mitochondrial candidates for follow-up experiments (53).

### 4.8 Functional enrichment analysis

We performed gene set enrichment analysis on the prioritized genes using g:Profiler, testing for over-representation of Gene Ontology terms and curated pathways (54); results with adjusted *p* < 0.05 were considered significant (55). We specifically examined whether the prioritized genes were enriched in known mitochondrial functions or kidney-related pathways (56).

### 4.9 Patients and tissue samples

We enrolled 20 allogeneic kidney transplant recipients diagnosed with CAD at the First Affiliated Hospital of Nanjing Medical University. All CAD cases were pathologically confirmed to have renal allograft interstitial fibrosis based on nephrectomy or biopsy specimens of transplanted kidneys. Normal kidney tissues were obtained from patients undergoing surgical nephrectomy for renal tumors, with tissue samples collected at least 5 cm away from the tumor margin to avoid tumor-associated alterations. All study protocols were approved by the Ethics Committee of the First Affiliated Hospital with Nanjing Medical University and were conducted in accordance with the Declaration of Helsinki and the Declaration of Istanbul. Written informed consent was obtained from all participants. Demographic and clinical characteristics of participants in the Normal and CAD groups are summarized in **Table 2**.

**Table 2:** Baseline characteristics of the normal and CAD groups.

Clinical variables	Normal group	CAD group	p-value
Case number (n)	20	20	>0.05
Gender (male/female)	14/6	12/8	>0.05
Age (years, mean ± SEM)	65.15 ± 1.36	59.60 ± 2.44	>0.05
BMI (kg/m², mean ± SEM)	22.77 ± 0.36	23.51 ± 0.38	>0.05
Transplant duration (years, mean ± SEM)	—	9.76 ± 0.42	—
Primary/secondary transplant	—	20/0	—
PRA before renal transplant (%)	—	0	—
Donor source			—
Living-related	—	4	—
Cadaveric	—	16	—
Immunosuppressive regimen			—
Prednisone + MMF + Tac	—	15	—
Prednisone + MMF + CsA	—	5	—
Serum creatinine (μmol/L, mean ± SEM)	70.55 ± 3.04	435.40 ± 8.43	<0.001

CAD, chronic allograft dysfunction; CsA, cyclosporine A; MMF, mycophenolate mofetil; PRA, panel reactive antibody; SEM, standard error of the mean; Tac, tacrolimus.

### 4.10 Animals

C57BL/6 and BALB/C mice were sourced from the Animal Center of Nanjing Medical University. All animals were housed in the specific pathogen-free Laboratory Animal Center at Nanjing Medical University.

### 4.11 Renal allogeneic transplantation mouse model

All animal procedures were approved by the Animal Research Ethics Committee of Nanjing Medical University. Renal allogeneic transplantation was performed as previously described with minor modifications. Briefly, mice were anesthetized with isoflurane inhalation (RWD Life Science). The right native kidney of the recipient mouse was excised *in situ*. Subsequently, the left kidney from a donor mouse was harvested and orthotopically transplanted into the recipient. The total ischemia time during surgery ranged from 40 to 60 minutes.

BALB/C mice were used as recipients. BALB/C donor kidneys were used to establish the Syn group, whereas C57BL/6 donor kidneys were used to generate the Allo group, which developed chronic renal allograft interstitial fibrosis. Contralateral nephrectomy of the recipient mice was performed 7 days after transplantation.

Tacrolimus (Astellas Pharmaceutical) was administered intraperitoneally at 1.5 mg/kg once daily for the first 7 days after transplantation, followed by 1.5 mg/kg once weekly for the subsequent 4 weeks. At 16 weeks post-transplantation, mice were euthanized, and transplanted kidneys were harvested, fixed in paraffin, or snap-frozen in liquid nitrogen for further analyses (57).

### 4.12 Cell culture and treatment

Human proximal tubular epithelial cells (HK-2) were obtained from the Cell Bank of the Typical Culture Preservation Committee, Chinese Academy of Sciences (Cat. No. SCSP-511). Cells were cultured in Dulbecco’s modified Eagle medium/Ham’s F-12 medium (DMEM/F-12) supplemented with 10% (v/v) fetal bovine serum (FBS; AusGeneX, Australia) and 1% (v/v) penicillin–streptomycin (Gibco, USA). All cells were maintained in a humidified incubator at 37 °C with 5% CO_2_. To induce a profibrotic phenotype, HK-2 cells were treated with recombinant human transforming growth factor-β (TGF-β; Novoprotein, GMP-CA59, 100 ng/mL) for 48h. Control cells were cultured under identical conditions in the absence of TGF-β (58).

### 4.13 RNA extraction and real-time quantitative PCR

Total RNA was isolated from cells or renal tissues using the RNAQuick Purification Kit (Shanghai YISHAN Biotechnology, RN001). Complementary DNA was synthesized using the HiScript II Q RT SuperMix (Vazyme, R222-01). RT-qPCR was performed using ChamQ SYBR qPCR Master Mix (Vazyme, Q341-02) on a StepOnePlus Real-Time PCR System (Applied Biosystems). Relative gene expression levels were calculated using the 2^−ΔΔCt^ method and normalized to GAPDH. Primer sequences used in this study are listed in **Supplementary Table 2**.

### 4.14 Plasmids, small interfering RNA and reagents

DNAs encoding human (h) GRIM-19 was inserted into the pFLAG-CMV-5a vector as well as DNA encoding GRIM-19 (accession no. NM_015965.6). All the constructed plasmids were confirmed by DNA sequencing. The plasmids transfection was performed using Lipofectamine 3000 (Invitrogen, L3000008). The siRNA targeting NDUFA13 (siNDUFA13) was synthesized by Shanghai GenePharma Co., Ltd., with the following sequence: 5′-GGAUUGGAACCCUGAUCUATT-3′ (59).

### 4.15 Western blotting

Cells or tissues were lysed using lysis buffer (Fdbio Science, FD009) supplemented with 1 mM PMSF (FD0100) and protease/phosphatase inhibitor cocktail (Thermo Fisher Scientific, 78442). Mitochondrial proteins were isolated using a mitochondrial protein extraction kit (BestBio). Protein concentration was determined using a BCA protein assay kit (Beyotime, P0010). Proteins were separated by SDS–PAGE and transferred onto PVDF membranes (Millipore, ISEQ00010). Membranes were blocked with 5% nonfat milk for 2 h at room temperature and incubated with primary antibodies at 4 °C overnight, followed by HRP-conjugated secondary antibodies for 2 h at room temperature. Protein bands were visualized using ECL reagents (Biosharp, BL520A). Antibodies used are listed in **Supplementary Table 3**.

### 4.16 Renal histopathological staining

Paraffin-embedded renal tissue sections were subjected to hematoxylin and eosin (HE) staining and Masson trichrome staining according to standard protocols. HE staining was used to evaluate renal structural alterations, while Masson staining was employed to assess the extent of renal interstitial fibrosis.

### 4.17 Immunohistochemistry and immunofluorescence staining

For IHC, paraffin-embedded sections were processed using an HRP detection kit (CWbio, CW2069S). For immunofluorescence (IF) staining, sections were baked at 65 °C for 2 h, deparaffinized in xylene, rehydrated through graded ethanol solutions, and subjected to antigen retrieval using enhanced citrate buffer (Beyotime, P0083). After blocking, sections were incubated with primary antibodies overnight at 4 °C, followed by appropriate fluorescent or HRP-conjugated secondary antibodies. For mitochondrial visualization, cells were incubated with MitoTracker™ Green FM (Thermo Fisher Scientific, Cat. No. A66441). Nuclei were counterstained with DAPI (SouthernBiotech, Cat. NO. 0100-20). Images were captured using fluorescence or confocal microscopy.

### 4.18 Mitochondrial membrane potential assay and ATP measurement

For mitochondrial membrane potential (Δψm) assessment, treated HK-2 cells were loaded with the potentiometric dye TMRE (500 nM; Beyotime, C2001S) at 37 °C for 20 min, washed, and imaged by confocal microscopy. TMRE fluorescence intensity was quantified from images to compare relative Δψm across conditions. Intracellular ATP levels were measured using an Enhanced ATP Assay Kit (Beyotime Biotechnology; Cat. No. S0027) following the manufacturer’s protocol, and ATP content was normalized to total protein concentration (60).

### 4.19 Measurement of mitochondrial superoxide production

To evaluate mitochondrial-specific superoxide production, HK-2 cells were stained using the MitoSOX™ Red Mitochondrial Superoxide Indicator (Beyotime, S0061S) according to the manufacturer’s instructions. Briefly, following the indicated treatments and transfections, cells were washed with Hank’s Balanced Salt Solution (HBSS) and incubated with 5 μM MitoSOX reagent at 37 °C in the dark for 30 min. After incubation, cells were washed three times with warm HBSS to remove excess unconjugated dye. Nuclei were counterstained with DAPI (SouthernBiotech, Cat. NO. 0100-20). Images were captured using fluorescence microscopy. The relative red fluorescence intensity of MitoSOX, representing mitochondrial superoxide levels, was quantified using ImageJ software (NIH, Bethesda, MD, USA). At least five randomly selected fields per experimental group were analyzed.

### 4.20 Transmission electron microscopy analysis

For transmission electron microscopy (TEM), cells were fixed in 2.5% glutaraldehyde at 4 °C overnight and post-fixed in 1% osmium tetroxide. Samples were dehydrated through graded ethanol solutions, embedded in epoxy resin, and sectioned into ultrathin slices. Sections were stained with uranyl acetate and lead citrate. Ultrastructural imaging was performed using a transmission electron microscope (FEI Tecnai G2) at the Analysis and Testing Center of Nanjing Medical University. Mitochondrial morphology, including cristae integrity and swelling, was evaluated.

### 4.21 Statistical analysis

All quantitative data are presented as the mean ± standard error of the mean (SEM). Each experiment was independently repeated at least three times. Statistical analyses were performed using GraphPad Prism software (version 8.0). For comparisons between two groups, Student’s t-test was applied. For comparisons involving more than two groups, one-way analysis of variance (ANOVA) followed by Dunnett’s or Tukey’s *post hoc* tests, as appropriate, was used. A *P* value < 0.05 was considered statistically significant.

## Data Availability

The datasets presented in this study can be found in online repositories. The names of the repository/repositories and accession number(s) can be found in the article/**Supplementary Material**.

## Glossary

ESRD: End-stage renal disease
MR: Randomization
IRI: ischemia-reperfusion injury
ROS: reactive oxygen species
mtDAMPs: mitochondrial damage-associated molecular patterns
mQTL: DNA methylation QTLs
eQTL: gene expression QTLs
pQTL: protein abundance QTLs
GWAS: genome-wide association study
SNP: single nucleotide polymorphism
SMR: summary-data based MR
FDR: false discovery rate
IHC: immunohistochemistry
IF: immunofluorescence
RT-qPCR: real-time quantitative PCR
Allo: allogeneic
Syn: Syngeneic
CAD: chronic allograft dysfunction
FN: Fibronectin
α-SMA: Alpha smooth muscle actin
E-Cad: E-Cadherin
Tom20: Translocase of Outer Mitochondrial Membrane 20
SOD2: Superoxide Dismutase 2
MTIF3: Mitochondrial translational initiation factor 3
MGME1: Mitochondrial Genome Maintenance Exonuclease 1
NDUFA13: NADH: Ubiquinone Oxidoreductase Subunit A13
MRPS18C: Mitochondrial Ribosomal Protein S18C

## References

[1] Menke J Sollinger D Schamberger B Heemann U Lutz J . The effect of ischemia/reperfusion on the kidney graft. Curr Opin Organ Transplant. (2014) 19:395–400. doi: 10.1097/mot.0000000000000090. 24905021

[2] Hart A Lentine KL Smith JM Miller JM Skeans MA Prentice M . OPTN/SRTR 2019 annual data report: kidney. Am J Transplant. (2021) 21:21–137. doi: 10.1111/ajt.16502, PMID: 33595191

[3] Boor P Floege J . Renal allograft fibrosis: biology and therapeutic targets. Am J Transplant. (2015) 15:863–86. doi: 10.1111/ajt.13180. 25691290

[4] Lobb I Davison M Carter D Liu W Haig A Gunaratnam L . Hydrogen sulfide treatment mitigates renal allograft ischemia-reperfusion injury during cold storage and improves early transplant kidney function and survival following allogeneic renal transplantation. J Urol. (2015) 194:1806–15. doi: 10.1016/j.juro.2015.07.096. 26239336

[5] Ponticelli C . Ischaemia-reperfusion injury: a major protagonist in kidney transplantation. Nephrol Dial Transplant. (2014) 29:1134–40. doi: 10.1093/ndt/gft488. 24335382

[6] Hayashida K Takegawa R Shoaib M Aoki T Choudhary RC Kuschner CE . Mitochondrial transplantation therapy for ischemia reperfusion injury: a systematic review of animal and human studies. J Transl Med. (2021) 19:214. doi: 10.1186/s12967-021-02878-3. 34001191 PMC8130169

[7] Niemann S Müller U . Mutations in SDHC cause autosomal dominant paraganglioma, type 3. Nat Genet. (2000) 26:268–70. doi: 10.1038/81551. 11062460

[8] Han F Wu S Dong Y Liu Y Sun B Chen L . Aberrant expression of NEDD4L disrupts mitochondrial homeostasis by downregulating CaMKKβ in diabetic kidney disease. J Transl Med. (2024) 22:465. doi: 10.1186/s12967-024-05207-6. 38755664 PMC11100153

[9] Zhao M Wang Y Li L Liu S Wang C Yuan Y . Mitochondrial ROS promote mitochondrial dysfunction and inflammation in ischemic acute kidney injury by disrupting TFAM-mediated mtDNA maintenance. Theranostics. (2021) 11:1845–63. doi: 10.7150/thno.50905. 33408785 PMC7778599

[10] Tao M You CP Zhao RR Liu SJ Zhang ZH Zhang C . Animal mitochondria: evolution, function, and disease. Curr Mol Med. (2014) 14:115–24. doi: 10.2174/15665240113136660081. 24195633

[11] Panconesi R Widmer J Carvalho MF Eden J Dondossola D Dutkowski P . Mitochondria and ischemia reperfusion injury. Curr Opin Organ Transplant. (2022) 27:434–45. doi: 10.1097/mot.0000000000001015. 35950880

[12] Dare AJ Bolton EA Pettigrew GJ Bradley JA Saeb-Parsy K Murphy MP . Protection against renal ischemia-reperfusion injury *in vivo* by the mitochondria targeted antioxidant MitoQ. Redox Biol. (2015) 5:163–8. doi: 10.1016/j.redox.2015.04.008, PMID: 25965144 PMC4427662

[13] Li Y Wang HB Cao JL Zhang WJ Wang HL Xu CH . Proteomic analysis of mitochondria associated membranes in renal ischemic reperfusion injury. J Transl Med. (2024) 22:261. doi: 10.1186/s12967-024-05021-0. 38461333 PMC10925013

[14] Jesinkey SR Funk JA Stallons LJ Wills LP Megyesi JK Beeson CC . Formoterol restores mitochondrial and renal function after ischemia-reperfusion injury. J Am Soc Nephrol. (2014) 25:1157–62. doi: 10.1681/asn.2013090952. 24511124 PMC4033382

[15] Birk AV Liu S Soong Y Mills W Singh P Warren JD . The mitochondrial-targeted compound SS-31 re-energizes ischemic mitochondria by interacting with cardiolipin. J Am Soc Nephrol. (2013) 24:1250–61. doi: 10.1681/ASN.2012121216, PMID: 23813215 PMC3736700

[16] Jabbari H Roushandeh AM Rostami MK Razavi-Toosi MT Shokrgozar MA Jahanian-Najafabadi A . Mitochondrial transplantation ameliorates ischemia/reperfusion-induced kidney injury in rat. Biochim Biophys Acta Mol Basis Dis. (2020) 1866:165809. doi: 10.1016/j.bbadis.2020.165809. 32353613

[17] Oka T Hikoso S Yamaguchi O Taneike M Takeda T Tamai T . Mitochondrial DNA that escapes from autophagy causes inflammation and heart failure. Nature. (2012) 485:251–5. doi: 10.1038/nature10992. 22535248 PMC3378041

[18] Feng J Chen Z Liang W Wei Z Ding G . Roles of mitochondrial DNA damage in kidney diseases: a new biomarker. Int J Mol Sci. (2022) 23(23):15166. doi: 10.3390/ijms232315166. 36499488 PMC9735745

[19] Jansen MPB Pulskens WP Butter LM Florquin S Juffermans NP Roelofs J . Mitochondrial DNA is released in urine of SIRS patients with acute kidney injury and correlates with severity of renal dysfunction. Shock. (2018) 49:301–10. doi: 10.1097/shk.0000000000000967. 28837526

[20] Kim K Moon H Lee YH Seo JW Kim YG Moon JY . Clinical relevance of cell-free mitochondrial DNA during the early postoperative period in kidney transplant recipients. Sci Rep. (2019) 9:18607. doi: 10.1038/s41598-019-54694-x. 31819080 PMC6901568

[21] Nakazawa D Kumar SV Marschner J Desai J Holderied A Rath L . Histones and neutrophil extracellular traps enhance tubular necrosis and remote organ injury in ischemic AKI. J Am Soc Nephrol. (2017) 28:1753–68. doi: 10.1681/asn.2016080925. 28073931 PMC5461800

[22] Dobrijevic E van Zwieten A Kiryluk K Grant AJ Wong G Teixeira-Pinto A . Mendelian randomization for nephrologists. Kidney Int. (2023) 104:1113–23. doi: 10.1016/j.kint.2023.09.016. 37783446

[23] Porcu E Rüeger S Lepik K Santoni FA Reymond A Kutalik Z . Mendelian randomization integrating GWAS and eQTL data reveals genetic determinants of complex and clinical traits. Nat Commun. (2019) 10:3300. doi: 10.1038/s41467-019-10936-0, PMID: 31341166 PMC6656778

[24] Sekula P Del Greco MF Pattaro C Köttgen A . Mendelian randomization as an approach to assess causality using observational data. J Am Soc Nephrol. (2016) 27:3253–65. doi: 10.1681/asn.2016010098. 27486138 PMC5084898

[25] Zheng J Zhang Y Zhao H Liu Y Baird D Karim MA . Multi-ancestry Mendelian randomization of omics traits revealing drug targets of COVID-19 severity. EBioMedicine. (2022) 81:104112. doi: 10.1016/j.ebiom.2022.104112. 35772218 PMC9235320

[26] Richardson TG Hemani G Gaunt TR Relton CL Davey Smith G . A transcriptome-wide Mendelian randomization study to uncover tissue-dependent regulatory mechanisms across the human phenome. Nat Commun. (2020) 11:185. doi: 10.1038/s41467-019-13921-9. 31924771 PMC6954187

[27] Zhou D Jiang Y Zhong X Cox NJ Liu C Gamazon ER . A unified framework for joint-tissue transcriptome-wide association and Mendelian randomization analysis. Nat Genet. (2020) 52:1239–46. doi: 10.1038/s41588-020-0706-2. 33020666 PMC7606598

[28] Rudler DL Hughes LA Perks KL Richman TR Kuznetsova I Ermer JA . Fidelity of translation initiation is required for coordinated respiratory complex assembly. Sci Adv. (2019) 5:eaay2118. doi: 10.1126/sciadv.aay2118. 31903419 PMC6924987

[29] Stroud DA Surgenor EE Formosa LE Reljic B Frazier AE Dibley MG . Accessory subunits are integral for assembly and function of human mitochondrial complex I. Nature. (2016) 538:123–6. doi: 10.1038/nature19754. 27626371

[30] Li L Zhang L Cao Y Chen X Gong H Ma Y . NDUFV1 attenuates renal ischemia-reperfusion injury by improving mitochondrial homeostasis. J Cell Mol Med. (2023) 27:1341–52. doi: 10.1111/jcmm.17735. 37029501 PMC10183703

[31] Chouchani ET Pell VR Gaude E Aksentijević D Sundier SY Robb EL . Ischaemic accumulation of succinate controls reperfusion injury through mitochondrial ROS. Nature. (2014) 515:431–5. doi: 10.1038/nature13909. 25383517 PMC4255242

[32] Verbanck M Chen CY Neale B Do R . Detection of widespread horizontal pleiotropy in causal relationships inferred from Mendelian randomization between complex traits and diseases. Nat Genet. (2018) 50:693–8. doi: 10.1038/s41588-018-0099-7. 29686387 PMC6083837

[33] Wainberg M Sinnott-Armstrong N Mancuso N Barbeira AN Knowles DA Golan D . Opportunities and challenges for transcriptome-wide association studies. Nat Genet. (2019) 51:592–9. doi: 10.1038/s41588-019-0385-z. 30926968 PMC6777347

[34] Hu X Lv J Zhao Y Li X Qi W Wang X . Important regulatory role of mitophagy in diabetic microvascular complications. J Transl Med. (2025) 23:269. doi: 10.1186/s12967-025-06307-7. 40038741 PMC11877814

[35] Smith GD Ebrahim S . 'Mendelian randomization': can genetic epidemiology contribute to understanding environmental determinants of disease? Int J Epidemiol. (2003) 32:1–22. doi: 10.1093/ije/dyg070. 12689998

[36] Thornton ZA Andrews LJ Zhao H Zheng J Paternoster L Robinson JW . Brain multi-omic Mendelian randomisation to identify novel drug targets for gliomagenesis. Hum Mol Genet. (2025) 34:178–92. doi: 10.1093/hmg/ddae168. 39565278 PMC11780873

[37] Jin C Lee B Shen L Long Q . Integrating multi-omics summary data using a Mendelian randomization framework. Brief Bioinform. (2022) 23(6):bbac376. doi: 10.1093/bib/bbac376. 36094096 PMC9677504

[38] Rath S Sharma R Gupta R Ast T Chan C Durham TJ . MitoCarta3.0: an updated mitochondrial proteome now with sub-organelle localization and pathway annotations. Nucleic Acids Res. (2021) 49:D1541–7. doi: 10.1093/nar/gkaa1011. 33174596 PMC7778944

[39] Cañadas-Garre M Anderson K McGoldrick J Maxwell AP McKnight AJ . Genomic approaches in the search for molecular biomarkers in chronic kidney disease. J Transl Med. (2018) 16:292. doi: 10.1186/s12967-018-1664-7. 30359254 PMC6203198

[40] Zhu Z Zheng Z Zhang F Wu Y Trzaskowski M Maier R . Causal associations between risk factors and common diseases inferred from GWAS summary data. Nat Commun. (2018) 9:224. doi: 10.1038/s41467-017-02317-2. 29335400 PMC5768719

[41] McRae AF Marioni RE Shah S Yang J Powell JE Harris SE . Identification of 55,000 replicated DNA methylation QTL. Sci Rep. (2018) 8:17605. doi: 10.1038/s41598-018-35871-w. 30514905 PMC6279736

[42] Võsa U Claringbould A Westra HJ Bonder MJ Deelen P Zeng B . Large-scale cis- and trans-eQTL analyses identify thousands of genetic loci and polygenic scores that regulate blood gene expression. Nat Genet. (2021) 53:1300–10. doi: 10.1038/s41588-021-00913-z, PMID: 34475573 PMC8432599

[43] Sun BB Chiou J Traylor M Benner C Hsu Y Richardson TG . Plasma proteomic associations with genetics and health in the UK Biobank. Nature. (2023) 622(7982):329–38. doi: 10.1038/s41586-023-06592-6. 37794186 PMC10567551

[44] Zhu Z Zhang F Hu H Bakshi A Robinson MR Powell JE . Integration of summary data from GWAS and eQTL studies predicts complex trait gene targets. Nat Genet. (2016) 48:481–7. doi: 10.1038/ng.3538, PMID: 27019110

[45] Xue A Wu Y Zhu Z Zhang F Kemper KE Zheng Z . Genome-wide association analyses identify 143 risk variants and putative regulatory mechanisms for type 2 diabetes. Nat Commun. (2018) 9:2941. doi: 10.1038/s41467-018-04951-w. 30054458 PMC6063971

[46] Wu Y Zeng J Zhang F Zhu Z Qi T Zheng Z . Integrative analysis of omics summary data reveals putative mechanisms underlying complex traits. Nat Commun. (2018) 9:918. doi: 10.1038/s41467-018-03371-0. 29500431 PMC5834629

[47] Giambartolomei C Vukcevic D SChadt EE Franke L Hingorani AD Wallace C . Bayesian test for colocalisation between pairs of genetic association studies using summary statistics. PloS Genet. (2014) 10:e1004383. doi: 10.1371/journal.pgen.1004383. 24830394 PMC4022491

[48] Hormozdiari F van de Bunt M Segrè AV Li X Joo JWJ Bilow M . Colocalization of GWAS and eQTL signals detects target genes. Am J Hum Genet. (2016) 99:1245–60. doi: 10.1016/j.ajhg.2016.10.003. 27866706 PMC5142122

[49] Foley CN Staley JR Breen PG Sun BB Kirk PDW Burgess S . A fast and efficient colocalization algorithm for identifying shared genetic risk factors across multiple traits. Nat Commun. (2021) 12:764. doi: 10.1038/s41467-020-20885-8. 33536417 PMC7858636

[50] Zheng J Haberland V Baird D Walker V Haycock PC Hurle MR . Phenome-wide Mendelian randomization mapping the influence of the plasma proteome on complex diseases. Nat Genet. (2020) 52:1122–31. doi: 10.1038/s41588-020-0682-6. 32895551 PMC7610464

[51] Hormozdiari F Gazal S van de Geijn B Finucane HK Ju CJ Loh PR . Leveraging molecular quantitative trait loci to understand the genetic architecture of diseases and complex traits. Nat Genet. (2018) 50:1041–7. doi: 10.1038/s41588-018-0148-2. 29942083 PMC6030458

[52] Sun BB Kurki MI Foley CN Mechakra A Chen CY Marshall E . Genetic associations of protein-coding variants in human disease. Nature. (2022) 603:95–102. doi: 10.1038/s41586-022-04394-w. 35197637 PMC8891017

[53] Liu X Li YI Pritchard JK . Trans effects on gene expression can drive omnigenic inheritance. Cell. (2019) 177:1022–1034.e6. doi: 10.1016/j.cell.2019.04.014, PMID: 31051098 PMC6553491

[54] Reimand J Isserlin R Voisin V Kucera M Tannus-Lopes C Rostamianfar A . Pathway enrichment analysis and visualization of omics data using g:Profiler, GSEA, Cytoscape and EnrichmentMap. Nat Protoc. (2019) 14:482–517. doi: 10.1038/s41596-018-0103-9. 30664679 PMC6607905

[55] Kuleshov MV Jones MR Rouillard AD Fernandez NF Duan Q Wang Z . Enrichr: a comprehensive gene set enrichment analysis web server 2016 update. Nucleic Acids Res. (2016) 44:W90–7. doi: 10.1093/nar/gkw377. 27141961 PMC4987924

[56] Li Y Wu FX Ngom A . A review on machine learning principles for multi-view biological data integration. Brief Bioinform. (2018) 19:325–40. doi: 10.1093/bib/bbw113. 28011753

[57] Feng D Gui Z Xu Z Zhang J Ni B Wang Z . Rictor/mTORC2 signalling contributes to renal vascular endothelial-to-mesenchymal transition and renal allograft interstitial fibrosis by regulating BNIP3-mediated mitophagy. Clin Transl Med. (2024) 14:e1686. doi: 10.1002/ctm2.1686, PMID: 38769658 PMC11106512

[58] Zhang Y Zhang J Feng D Zhou H Gui Z Zheng M . IRF1/ZNF350/GPX4-mediated ferroptosis of renal tubular epithelial cells promote chronic renal allograft interstitial fibrosis. Free Radic Biol Med. (2022) 193:579–94. doi: 10.1016/j.freeradbiomed.2022.11.002. 36356714

[59] Jeong HY Park JS Choi JW Lee KH Yang SC Kang HY . GRIM-19-mediated induction of mitochondrial STAT3 alleviates systemic sclerosis by inhibiting fibrosis and Th2/Th17 cells. Exp Mol Med. (2024) 56:2739–46. doi: 10.1038/s12276-024-01366-0. 39643607 PMC11671530

[60] Su Z Li J Lin J Li Z Che Y Zhang Z . TNF-α-induced KAT2A impedes BMMSC quiescence by mediating succinylation of the mitophagy-related protein VCP. Adv Sci Weinh. (2024) 11:e2303388. doi: 10.1002/advs.202303388. 38145956 PMC10933659

