# Supplementary material for: Genetically prioritized mitochondrial regulators of advanced renal failure: multi-omic Mendelian randomization and biological plausibility assessment in allograft fibrosis
Source: Front Immunol. 2026 Mar 27;17:1783844. doi: 10.3389/fimmu.2026.1783844 (PMC13065693; doi:10.3389/fimmu.2026.1783844)
Supplement: Supplementary file 1 [file DataSheet1.pdf]

Supplementary Material

1.1 Supplementary Figures

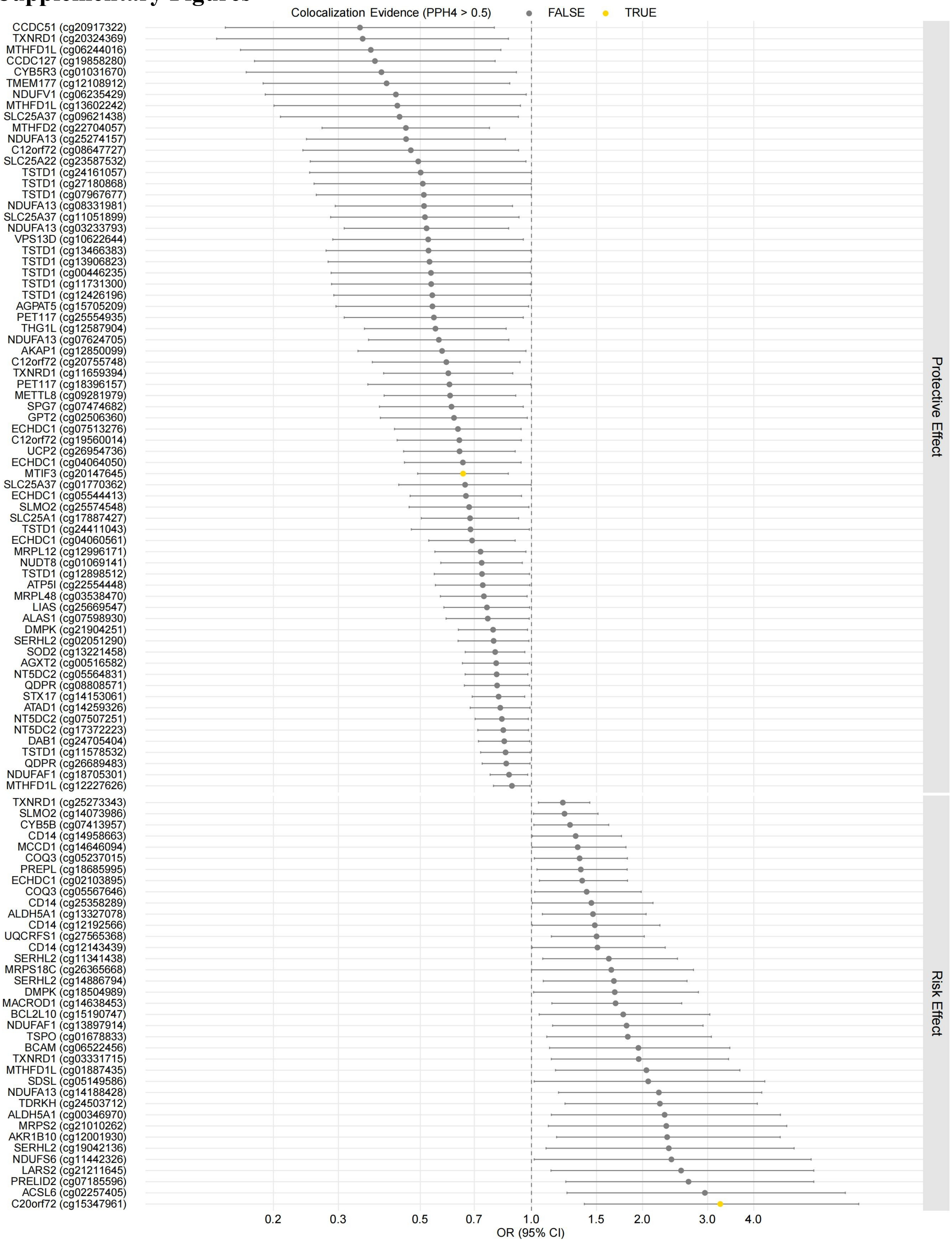

Supplementary Figure 1. Forest plot of mQTL-based Mendelian randomization results for mitochondrial CpG sites associated with kidney ESRD risk. This plot shows the causal associations between DNA methylation at mitochondrial gene CpG loci and kidney ESRD risk, using mQTL instruments. For each CpG, the odds ratio (OR) and 95% confidence interval for ESRD risk are plotted, grouped by whether higher methylation is protective (OR < 1) or risk-increasing (OR > 1). CpG sites with at least suggestive colocalisation evidence (PPH4 > 0.50) are highlighted in the plot. Statistical significance was defined as FDR < 0.05; colocalisation was defined as PP_H4 ≥ 0.70 (suggestive: 0.50 ≤ PPH4 < 0.70).

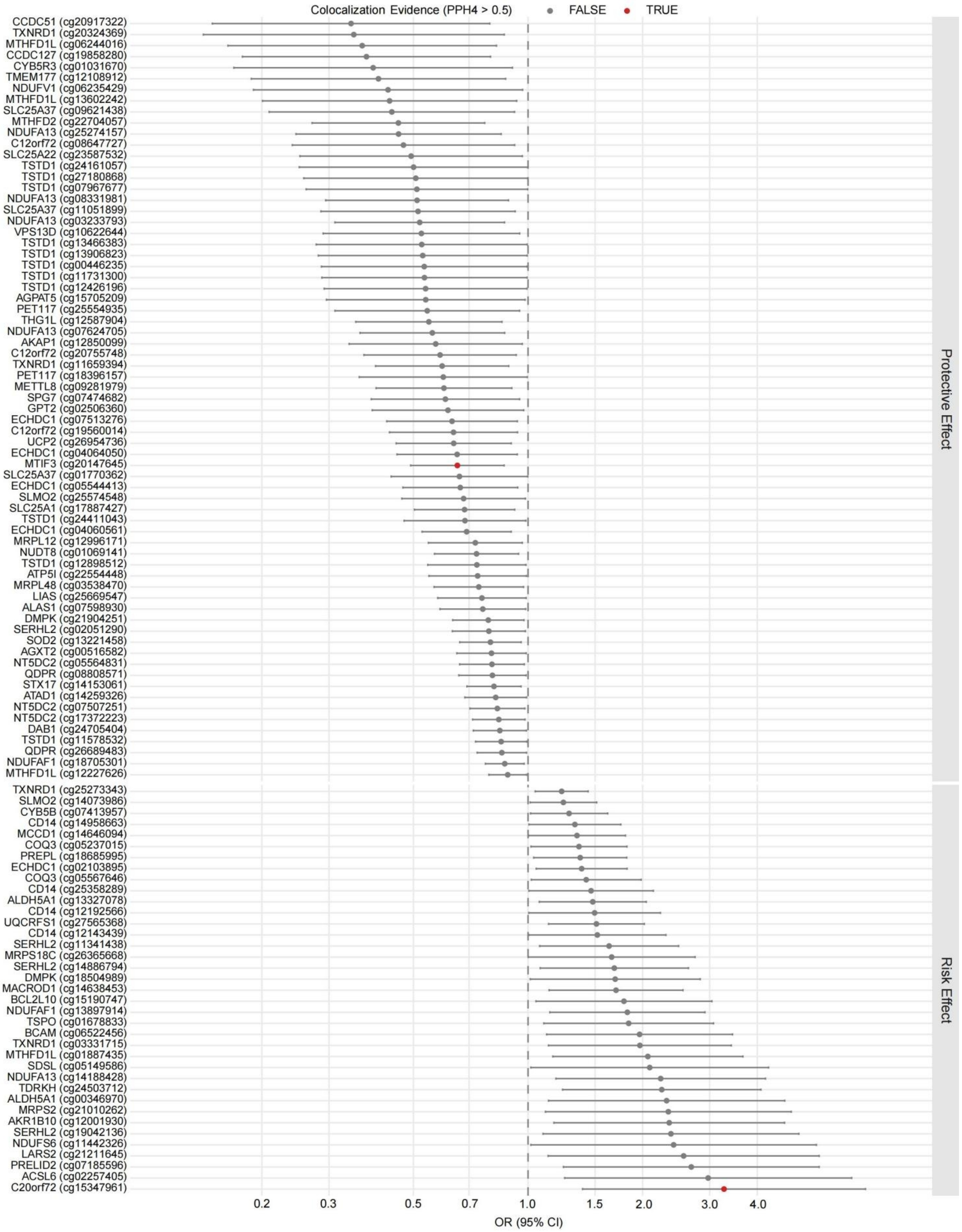
Colocalization Evidence (PPH4 > 0.5)
FALSE
TRUE
CCDC51 (cg20917322)
TXNRD1 (cg20324369)
MTHFD1L (cg06244016)
CCDC127 (cg19858280)
CYB5R3 (cg01031670)
TMEM177 (cg12108912)
NDUFV1 (cg06235429)
MTHFD1L (cg13602242)
SLC25A37 (cg09621438)
MTHFD2 (cg22704057)
NDUFA13 (cg25274157)
C12orf72 (cg08647727)
SLC25A22 (cg23587532)
TSTD1 (cg24161057)
TSTD1 (cg27180868)
TSTD1 (cg07967677)
NDUFA13 (cg08331981)
SLC25A37 (cg11051899)
NDUFA13 (cg03233793)
VPS13D (cg10622644)
TSTD1 (cg13466383)
TSTD1 (cg13906823)
TSTD1 (cg00446235)
TSTD1 (cg11731300)
TSTD1 (cg12426196)
AGPAT5 (cg15705209)
PET117 (cg25554935)
THG1L (cg12587904)
NDUFA13 (cg07624705)
AKAP1 (cg12850099)
C12orf72 (cg20755748)
TXNRD1 (cg11659394)
PET117 (cg18396157)
METTL8 (cg09281979)
SPG7 (cg07474682)
GPT2 (cg02506360)
ECHDC1 (cg07513276)
C12orf72 (cg19560014)
UCP2 (cg26954736)
ECHDC1 (cg04064050)
MTIF3 (cg20147645)
SLC25A37 (cg01770362)
ECHDC1 (cg05544413)
SLMO2 (cg25574548)
SLC25A1 (cg17887427)
TSTD1 (cg24411043)
ECHDC1 (cg04060561)
MRPL12 (cg12996171)
NUDT8 (cg01069141)
TSTD1 (cg12898512)
ATP5I (cg22554448)
MRPL48 (cg03538470)
LIAS (cg25669547)
ALAS1 (cg07598930)
DMPK (cg21904251)
SERHL2 (cg02051290)
SOD2 (cg13221458)
AGXT2 (cg00516582)
NT5DC2 (cg05564831)
QDPR (cg08808571)
STX17 (cg14153061)
ATAD1 (cg14259326)
NT5DC2 (cg07507251)
NT5DC2 (cg17372223)
DAB1 (cg24705404)
TSTD1 (cg11578532)
QDPR (cg26689483)
NDUFAF1 (cg18705301)
MTHFD1L (cg12227626)
TXNRD1 (cg25273343)
SLMO2 (cg14073986)
CYB5B (cg07413957)
CD14 (cg14958663)
MCCD1 (cg14646094)
COQ3 (cg05237015)
PREPL (cg18685995)
ECHDC1 (cg02103895)
COQ3 (cg05567646)
CD14 (cg25358289)
ALDH5A1 (cg13327078)
CD14 (cg12192566)
UQCRFS1 (cg27565368)
CD14 (cg12143439)
SERHL2 (cg11341438)
MRPS18C (cg26365668)
SERHL2 (cg14886794)
DMPK (cg18504989)
MACROD1 (cg14638453)
BCL2L10 (cg15190747)
NDUFAF1 (cg13897914)
TSPO (cg01678833)
BCAM (cg06522456)
TXNRD1 (cg03331715)
MTHFD1L (cg01887435)
SDSL (cg05149586)
NDUFA13 (cg14188428)
TDRKH (cg24503712)
ALDH5A1 (cg00346970)
MRPS2 (cg21010262)
AKR1B10 (cg12001930)
SERHL2 (cg19042136)
NDUFS6 (cg11442326)
LARS2 (cg21211645)
PRELID2 (cg07185596)
ACSL6 (cg02257405)
C20orf72 (cg15347961)
Protective Effect
Risk Effect
0.2
0.3
0.5
0.7
1.0
1.5
2.0
3.0
4.0
OR (95% CI)

Supplementary Figure 2. Forest plot of DNA methylation MR associations with ESRD risk (updated analysis). This updated plot presents the Mendelian randomization results for DNA methylation sites associated with kidney ESRD risk, with each CpG site labeled by its host gene. Entries are grouped by effect direction (protective vs. risk) as in Figure S1, and an indicator denotes whether each association has colocalisation support. **Statistical significance was defined as FDR < 0.05; colocalisation was defined as PP_H4 ≥ 0.70 (suggestive: 0.50 ≤ PPH4 < 0.70).**

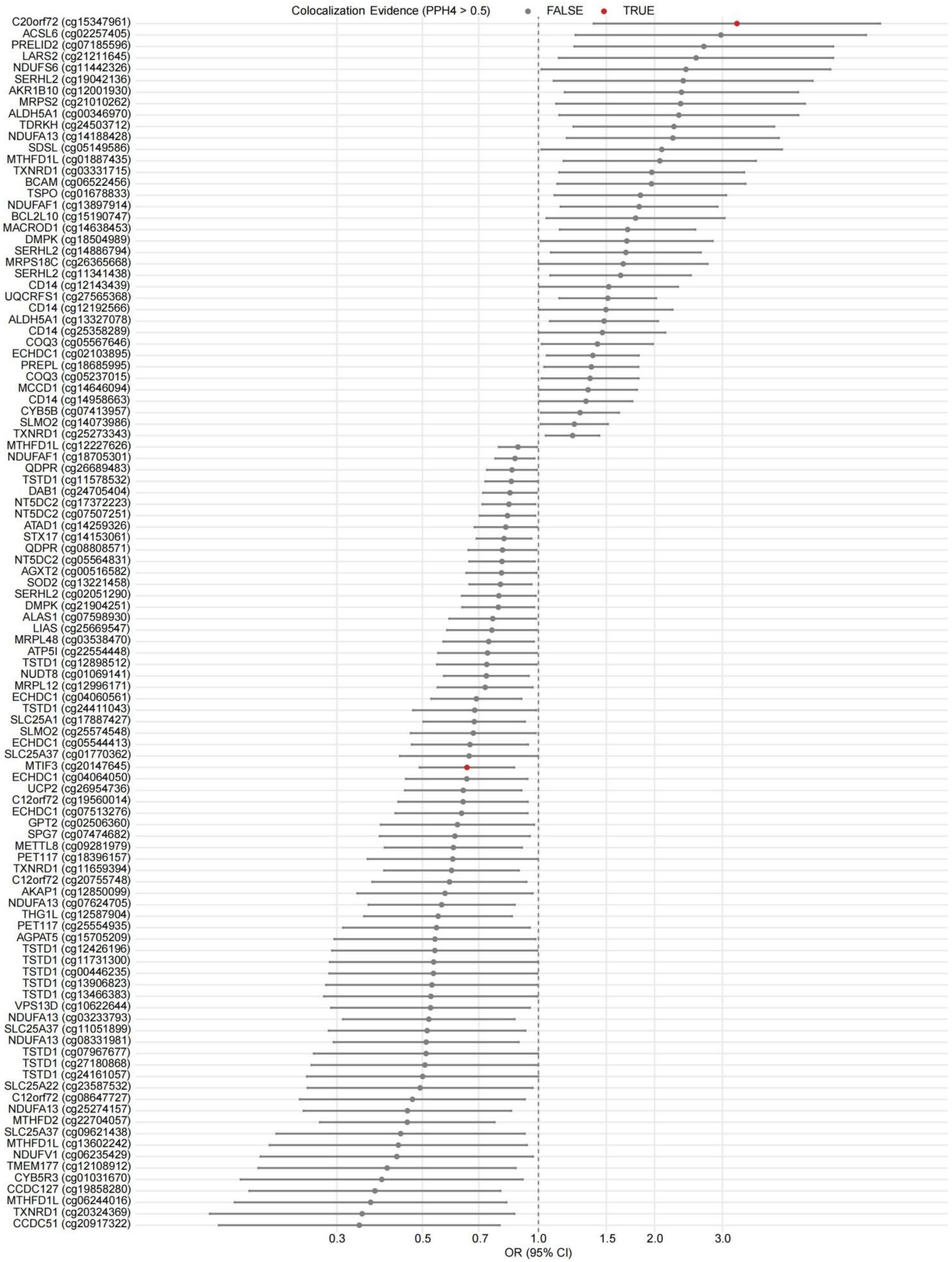
Colocalization Evidence (PPH4 > 0.5)
FALSE
TRUE
C20orf72 (cg15347961)
ACSL6 (cg02257405)
PRELID2 (cg07185596)
LARS2 (cg21211645)
NDUFS6 (cg11442326)
SERHL2 (cg19042136)
AKR1B10 (cg12001930)
MRPS2 (cg21010262)
ALDH5A1 (cg00346970)
TDRKH (cg24503712)
NDUFA13 (cg14188428)
SDSL (cg05149586)
MTHFD1L (cg01887435)
TXNRD1 (cg03331715)
BCAM (cg06522456)
TSPO (cg01678833)
NDUFAF1 (cg13897914)
BCL2L10 (cg15190747)
MACROD1 (cg14638453)
DMPK (cg18504989)
SERHL2 (cg14886794)
MRPS18C (cg26365668)
SERHL2 (cg11341438)
CD14 (cg12143439)
UQCRFS1 (cg27565368)
CD14 (cg12192566)
ALDH5A1 (cg13327078)
CD14 (cg25358289)
COQ3 (cg05567646)
ECHDC1 (cg02103895)
PREPL (cg18685995)
COQ3 (cg05237015)
MCCD1 (cg14646094)
CD14 (cg14958663)
CYB5B (cg07413957)
SLMO2 (cg14073986)
TXNRD1 (cg25273343)
MTHFD1L (cg12227626)
NDUFAF1 (cg18705301)
QDPR (cg26689483)
TSTD1 (cg11578532)
DAB1 (cg24705404)
NT5DC2 (cg17372223)
NT5DC2 (cg07507251)
ATAD1 (cg14259326)
STX17 (cg14153061)
QDPR (cg08808571)
NT5DC2 (cg05564831)
AGXT2 (cg00516582)
SOD2 (cg13221458)
SERHL2 (cg02051290)
DMPK (cg21904251)
ALAS1 (cg07598930)
LIAS (cg25669547)
MRPL48 (cg03538470)
ATP5I (cg22554448)
TSTD1 (cg12898512)
NUDT8 (cg01069141)
MRPL12 (cg12996171)
ECHDC1 (cg04060561)
TSTD1 (cg24411043)
SLC25A1 (cg17887427)
SLMO2 (cg25574548)
ECHDC1 (cg05544413)
SLC25A37 (cg01770362)
MTIF3 (cg20147645)
ECHDC1 (cg04064050)
UCP2 (cg26954736)
C12orf72 (cg19560014)
ECHDC1 (cg07513276)
GPT2 (cg02506360)
SPG7 (cg07474682)
METTL8 (cg09281979)
PET117 (cg18396157)
TXNRD1 (cg11659394)
C12orf72 (cg20755748)
AKAP1 (cg12850099)
NDUFA13 (cg07624705)
THG1L (cg12587904)
PET117 (cg25554935)
AGPAT5 (cg15705209)
TSTD1 (cg12426196)
TSTD1 (cg11731300)
TSTD1 (cg00446235)
TSTD1 (cg13906823)
TSTD1 (cg13466383)
VPS13D (cg10622644)
NDUFA13 (cg03233793)
SLC25A37 (cg11051899)
NDUFA13 (cg08331981)
TSTD1 (cg07967677)
TSTD1 (cg27180868)
TSTD1 (cg24161057)
SLC25A22 (cg23587532)
C12orf72 (cg08647727)
NDUFA13 (cg25274157)
MTHFD2 (cg22704057)
SLC25A37 (cg09621438)
MTHFD1L (cg13602242)
NDUFV1 (cg06235429)
TMEM177 (cg12108912)
CYB5R3 (cg01031670)
CCDC127 (cg19858280)
MTHFD1L (cg06244016)
TXNRD1 (cg20324369)
CCDC51 (cg20917322)
0.3
0.5
0.7
1.0
1.5
2.0
3.0
OR (95% CI)

Supplementary Figure 3.Condensed summary of top methylation–ESRD associations. A streamlined forest plot highlights the most robustly associated mitochondrial CpG sites with ESRD risk, restricted to those with colocalisation support. This visualization emphasizes the highest-confidence (Tier 1) candidates and their consistent effect directions (protective or risk-increasing). **Statistical significance was defined as FDR < 0.05; colocalisation was defined as PPH4 ≥ 0.70 (suggestive: 0.50 ≤ PPH4 < 0.70).**

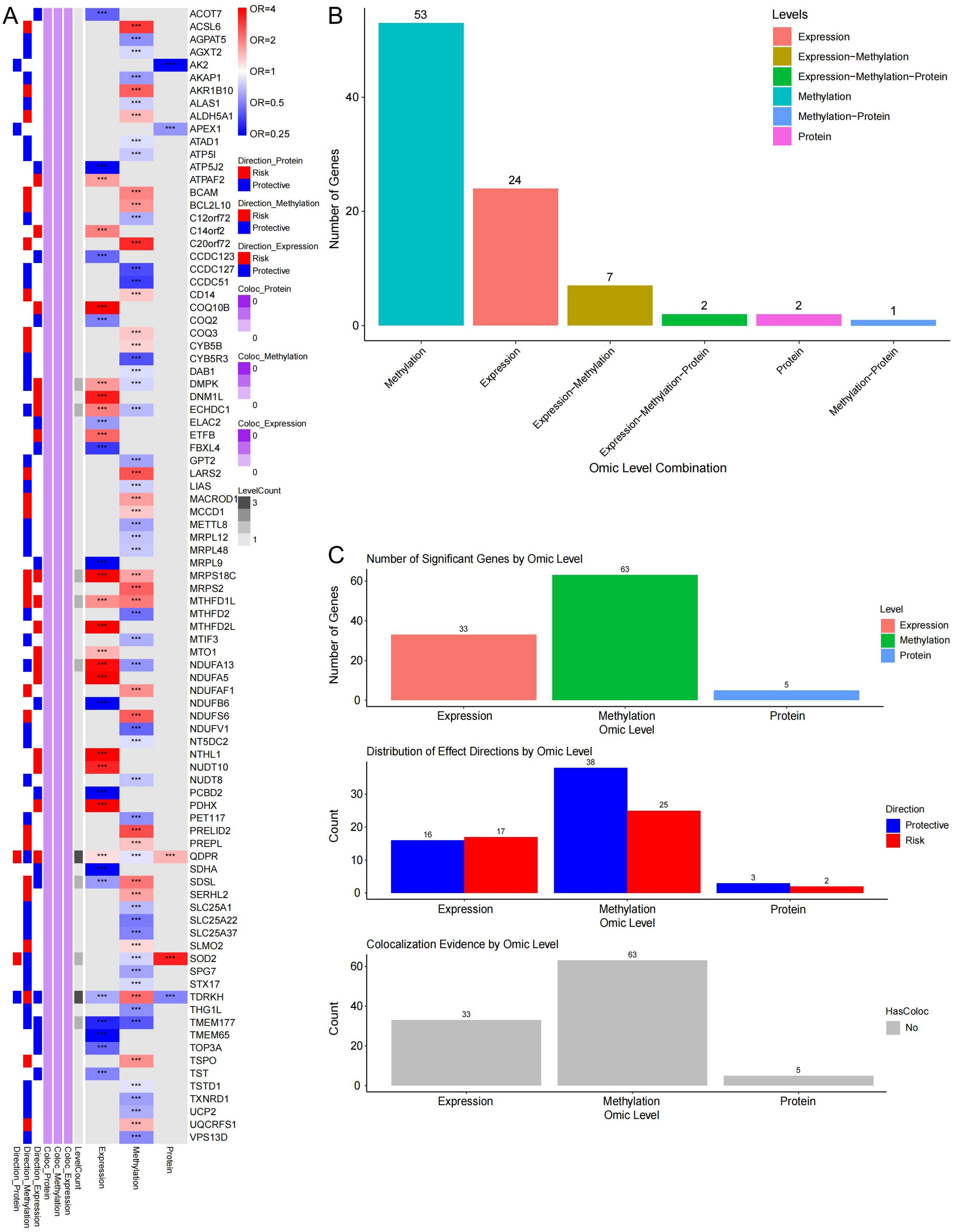

Supplementary Figure 4. Overview of multi-omic MR results across all mitochondrial genes. Bar plots summarize the significant associations identified by MR across the three molecular layers.

(A) Number of mitochondrial genes yielding significant causal associations in each omics layer (gene expression, DNA methylation, and protein abundance). (B) Proportion of significant associations with protective effects versus risk-increasing effects in each layer. (C) Proportion of significant associations with strong colocalisation support ($PPH4 \geq 0.70$) in each layer. **Statistical significance was defined as $FDR < 0.05$; colocalisation was defined as $PPH4 \geq 0.70$ (suggestive: $0.50 \leq PPH4 < 0.70$).**

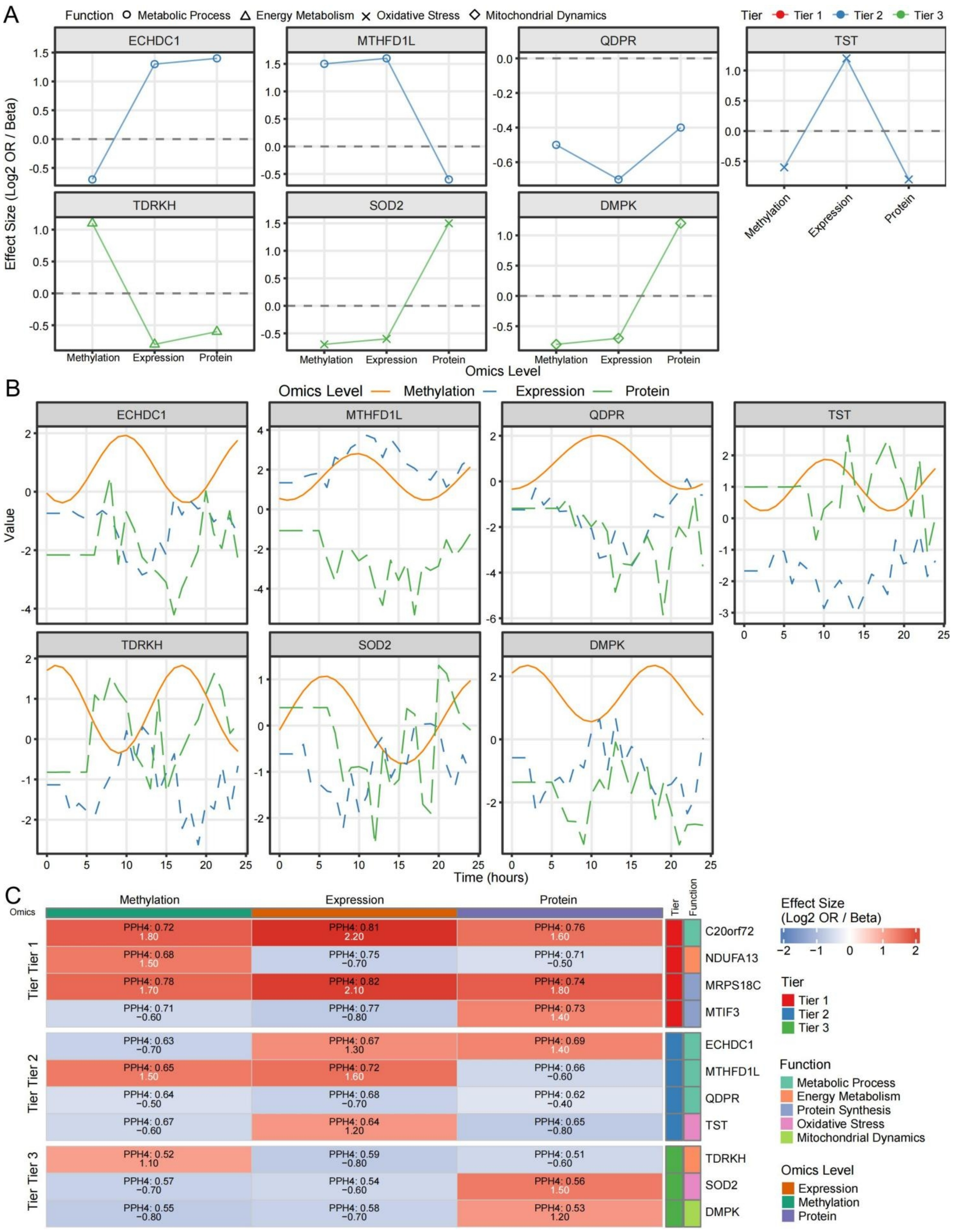

Supplementary Figure 5. Effect sizes and cross-omic regulatory consistency for prioritized genes. (A) Effect sizes of each prioritized gene's association with ESRD risk across the three omics

layers ($\log_2$ OR or β in the methylation, expression, and protein MR analyses). (B) Multi-parameter profiles for each prioritized gene, summarizing its functional category, pathway involvement, evidence tier (Tier 1 or 2), effect size strength, and colocalisation support. (C) Temporal consistency of regulatory effects for selected candidate genes, illustrating coherent trends across omics layers and the inferred latency (time lag) between molecular changes. **Statistical significance was defined as FDR < 0.05; colocalisation was defined as PPH4 ≥ 0.70 (suggestive: 0.50 ≤ PPH4 < 0.70).**

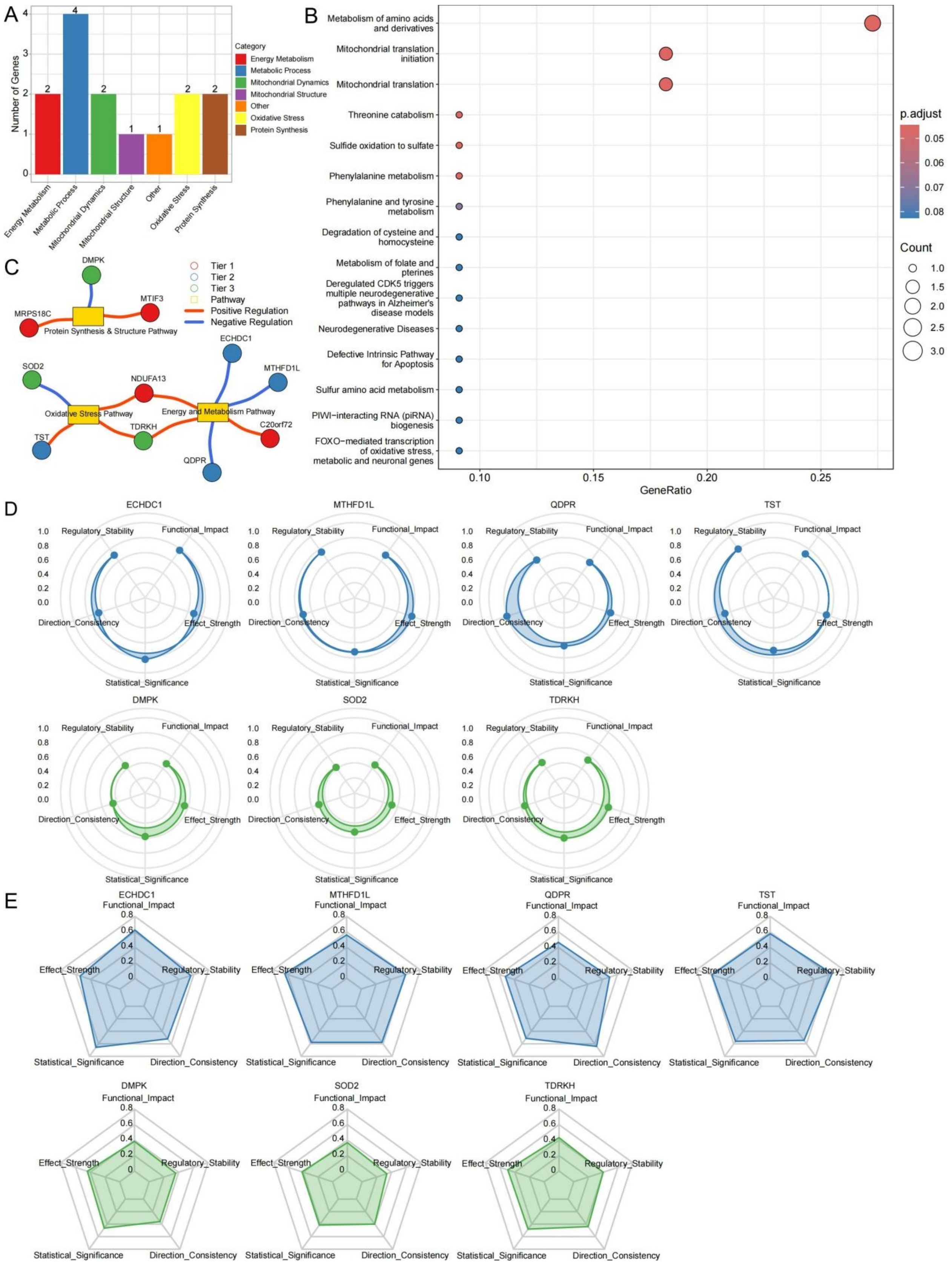

Supplementary Figure 6. Functional categorization and multi-domain prioritization of candidate genes. (A) Functional categories enriched among the candidate genes (Tiers 1–3), grouped

into key mitochondrial processes (e.g., energy metabolism, oxidative stress response, protein synthesis). (B) Pathway enrichment analysis for the top candidate genes, highlighting significantly overrepresented pathways from KEGG, Reactome, and Hallmark gene sets. (C) Mapping of candidate genes to their associated pathways, with coloring to indicate evidence tier (for genes) and pathway class. (D) Radar plots of five domain-specific scores for each top candidate gene (functional impact, effect size strength, statistical significance, direction consistency across layers, and regulatory stability). (E) Aggregated bar plots comparing the five domain scores across Tier 1, Tier 2, and Tier 3 groups, highlighting the strongest drivers of gene prioritization. **Statistical significance was defined as FDR < 0.05; colocalisation was defined as PPH4 ≥ 0.70 (suggestive: 0.50 ≤ PPH4 < 0.70).**

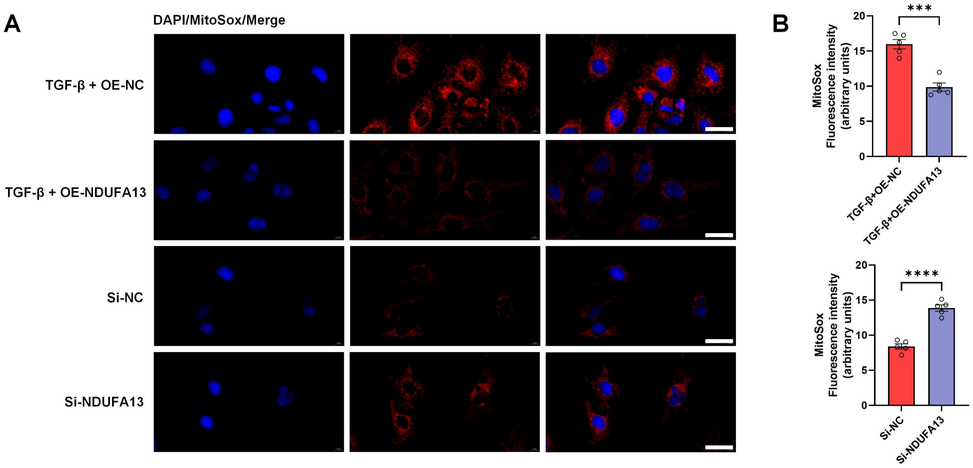

Supplementary Figure 7. *NDUFA13* regulates mitochondrial superoxide production in HK-2 cells. (A) Representative fluorescence micrographs of HK-2 cells stained with DAPI and MitoSOX Red under the indicated experimental conditions: TGF-β stimulation with empty vector (TGF-β + OE-NC), TGF-β stimulation with *NDUFA13* overexpression plasmid (TGF-β + OE-NDUFA13), transfection with *NDUFA13* siRNA without TGF-β stimulation (si-*NDUFA13*), and transfection with negative control siRNA without TGF-β stimulation (si-NC). Bar = 35 μm. (B) Quantitative analyses of the relative MitoSOX fluorescence intensity in the indicated groups (n = 5). ***$P < 0.001$, ****$P < 0.0001$.
