# Supplementary material for: Genetically prioritized mitochondrial regulators of advanced renal failure: multi-omic Mendelian randomization and biological plausibility assessment in allograft fibrosis
Source: Front Immunol. 2026 Mar 27;17:1783844. doi: 10.3389/fimmu.2026.1783844 (PMC13065693; doi:10.3389/fimmu.2026.1783844)
Supplement: Supplementary file 2 [file Table1.docx]

**Table S1. Additional mitochondrial genes with nominal associations not meeting multi-omic evidence thresholds (Tier 5, non-prioritized).**

| **Gene** | **Category** | **Methylation_Effect** **(OR [95% CI]; P-value)** | **Expression_Effect**  **(OR [95% CI]; P-value)** | **Protein_Effect**  **(OR [95% CI]; P-value)** | **Colocalization**  **(PP_H4: M / E / P)** | **Evidence_Tier** |
| --- | --- | --- | --- | --- | --- | --- |
| ACOT7 | NA | - | 0.43 (0.2-0.92), P=2.90e-02 | - | M:0, E:0.16, P:0 | 5 |
| ACSL6 | NA | 2.97 (1.25-7.08), P=1.39e-02 | - | - | M:0.42, E:0, P:0 | 5 |
| AGPAT5 | NA | 0.54 (0.3-0.98), P=4.35e-02 | - | - | M:0.13, E:0, P:0 | 5 |
| AGXT2 | NA | 0.8 (0.65-0.99), P=3.88e-02 | - | - | M:0.15, E:0, P:0 | 5 |
| AKAP1 | NA | 0.57 (0.34-0.97), P=3.67e-02 | - | - | M:0.16, E:0, P:0 | 5 |
| AKR1B10 | NA | 2.35 (1.17-4.72), P=1.64e-02 | - | - | M:0.23, E:0, P:0 | 5 |
| ALAS1 | NA | 0.76 (0.59-0.99), P=3.87e-02 | - | - | M:0.05, E:0, P:0 | 5 |
| ALDH5A1 | NA | 1.85 (1.1-3.11), P=1.77e-02 | - | - | M:0.26, E:0, P:0 | 5 |
| ATAD1 | NA | 0.82 (0.68-0.99), P=4.00e-02 | - | - | M:0.12, E:0, P:0 | 5 |
| ATP5I | NA | 0.74 (0.55-0.99), P=4.27e-02 | - | - | M:0.17, E:0, P:0 | 5 |
| ATP5J2 | NA | - | 0.14 (0.03-0.69), P=1.50e-02 | - | M:0, E:0.09, P:0 | 5 |
| ATPAF2 | NA | - | 1.66 (1.02-2.73), P=4.32e-02 | - | M:0, E:0.12, P:0 | 5 |
| BCAM | NA | 1.96 (1.12-3.44), P=1.86e-02 | - | - | M:0.22, E:0, P:0 | 5 |
| BCL2L10 | NA | 1.79 (1.05-3.04), P=3.26e-02 | - | - | M:0.13, E:0, P:0 | 5 |
| C12orf72 | NA | 0.56 (0.34-0.93), P=2.25e-02 | - | - | M:0.15, E:0, P:0 | 5 |
| C14orf2 | NA | - | 2.02 (1.01-4.07), P=4.83e-02 | - | M:0, E:0.09, P:0 | 5 |
| C20orf72 | Mitochondrial Function | 3.27 (1.39-7.7), P=6.62e-03 | - | - | M:0.55, E:0, P:0 | 5 |
| CCDC123 | NA | - | 0.43 (0.2-0.93), P=3.26e-02 | - | M:0, E:0.13, P:0 | 5 |
| CCDC127 | NA | 0.38 (0.18-0.8), P=1.07e-02 | - | - | M:0.42, E:0, P:0 | 5 |
| CCDC51 | NA | 0.34 (0.15-0.79), P=1.24e-02 | - | - | M:0.08, E:0, P:0 | 5 |
| CD14 | NA | 1.45 (1.01-2.09), P=4.55e-02 | - | - | M:0.14, E:0, P:0 | 5 |
| COQ10B | Electron Transport Chain | - | 20.9 (2.42-180.43), P=5.71e-03 | - | M:0, E:0.47, P:0 | 5 |
| COQ2 | NA | - | 0.5 (0.27-0.95), P=3.56e-02 | - | M:0, E:0.13, P:0 | 5 |
| COQ3 | NA | 1.39 (1.02-1.9), P=3.73e-02 | - | - | M:0.16, E:0, P:0 | 5 |
| CYB5B | NA | 1.28 (1.01-1.62), P=3.71e-02 | - | - | M:0.18, E:0, P:0 | 5 |
| CYB5R3 | NA | 0.39 (0.17-0.91), P=2.93e-02 | - | - | M:0.11, E:0, P:0 | 5 |
| DAB1 | NA | 0.84 (0.72-0.99), P=3.68e-02 | - | - | M:0.16, E:0, P:0 | 5 |
| DMPK | NA | 1.15 (0.8-1.66), P=2.89e-02 | 1.75 (1.05-2.93), P=3.26e-02 | - | M:0.15, E:0.12, P:0 | 5 |
| DNM1L | NA | - | 3.52 (1.25-9.9), P=1.70e-02 | - | M:0, E:0.19, P:0 | 5 |
| ECHDC1 | Lipid Metabolism | 0.76 (0.55-1.06), P=6.94e-03 | 2.03 (1.12-3.67), P=1.99e-02 | - | M:0.23, E:0.2, P:0 | 5 |
| ELAC2 | NA | - | 0.58 (0.38-0.88), P=1.10e-02 | - | M:0, E:0.25, P:0 | 5 |
| ETFB | NA | - | 2.32 (1.08-4.95), P=3.04e-02 | - | M:0, E:0.15, P:0 | 5 |
| FBXL4 | NA | - | 0.34 (0.12-0.98), P=4.61e-02 | - | M:0, E:0.1, P:0 | 5 |
| GPT2 | NA | 0.62 (0.39-0.97), P=3.83e-02 | - | - | M:0.19, E:0, P:0 | 5 |
| LARS2 | NA | 2.56 (1.13-5.82), P=2.44e-02 | - | - | M:0.19, E:0, P:0 | 5 |
| LIAS | NA | 0.76 (0.58-0.99), P=4.11e-02 | - | - | M:0.12, E:0, P:0 | 5 |
| MACROD1 | NA | 1.7 (1.14-2.55), P=9.99e-03 | - | - | M:0.31, E:0, P:0 | 5 |
| MCCD1 | NA | 1.34 (1-1.8), P=4.76e-02 | - | - | M:0.15, E:0, P:0 | 5 |
| METTL8 | NA | 0.6 (0.4-0.91), P=1.52e-02 | - | - | M:0.31, E:0, P:0 | 5 |
| MRPL12 | NA | 0.73 (0.55-0.97), P=2.78e-02 | - | - | M:0.21, E:0, P:0 | 5 |
| MRPL48 | NA | 0.74 (0.57-0.97), P=3.09e-02 | - | - | M:0.16, E:0, P:0 | 5 |
| MRPL9 | NA | - | 0.09 (0.01-0.69), P=2.07e-02 | - | M:0, E:0.16, P:0 | 5 |
| MRPS18C | Mitochondrial Ribosome | 1.66 (1-2.75), P=4.94e-02 | 4.11 (1.46-11.55), P=7.38e-03 | - | M:0.16, E:0.34, P:0 | 5 |
| MRPS2 | NA | 2.34 (1.11-4.91), P=2.52e-02 | - | - | M:0.18, E:0, P:0 | 5 |
| MTHFD1L | One-carbon Metabolism | 0.73 (0.42-1.29), P=1.35e-02 | 1.85 (1.04-3.29), P=3.64e-02 | - | M:0.31, E:0.11, P:0 | 5 |
| MTHFD2 | NA | 0.46 (0.27-0.77), P=3.24e-03 | - | - | M:0.48, E:0, P:0 | 5 |
| MTHFD2L | NA | - | 4.46 (1.13-17.62), P=3.31e-02 | - | M:0, E:0.13, P:0 | 5 |
| MTIF3 | Mitochondrial Translation | 0.65 (0.49-0.87), P=3.05e-03 | - | - | M:0.5, E:0, P:0 | 5 |
| MTO1 | NA | - | 1.49 (1.01-2.19), P=4.27e-02 | - | M:0, E:0.12, P:0 | 5 |
| NDUFA13 | NA | 0.69 (0.4-1.19), P=9.49e-03 | 3.81 (1.37-10.59), P=1.03e-02 | - | M:0.31, E:0.28, P:0 | 5 |
| NDUFA5 | NA | - | 15.97 (1.91-133.23), P=1.05e-02 | - | M:0, E:0.18, P:0 | 5 |
| NDUFAF1 | NA | 1.26 (0.94-1.69), P=1.20e-02 | - | - | M:0.35, E:0, P:0 | 5 |
| NDUFB6 | NA | - | 0.13 (0.02-0.8), P=2.80e-02 | - | M:0, E:0.13, P:0 | 5 |
| NDUFS6 | NA | 2.41 (1.02-5.72), P=4.56e-02 | - | - | M:0.16, E:0, P:0 | 5 |
| NDUFV1 | NA | 0.43 (0.19-0.97), P=4.15e-02 | - | - | M:0.23, E:0, P:0 | 5 |
| NT5DC2 | NA | 0.82 (0.69-0.98), P=2.85e-02 | - | - | M:0.2, E:0, P:0 | 5 |
| NTHL1 | NA | - | 5.12 (1.01-25.89), P=4.81e-02 | - | M:0, E:0.11, P:0 | 5 |
| NUDT10 | NA | - | 3.33 (1.39-7.99), P=6.99e-03 | - | M:0, E:0.33, P:0 | 5 |
| NUDT8 | NA | 0.73 (0.57-0.94), P=1.63e-02 | - | - | M:0.29, E:0, P:0 | 5 |
| PCBD2 | NA | - | 0.03 (0-0.57), P=1.93e-02 | - | M:0, E:0.17, P:0 | 5 |
| PDHX | Energy Metabolism | - | 4.92 (1.61-15.03), P=5.16e-03 | - | M:0, E:0.37, P:0 | 5 |
| PET117 | NA | 0.57 (0.33-0.97), P=3.24e-02 | - | - | M:0.1, E:0, P:0 | 5 |
| PRELID2 | NA | 2.68 (1.24-5.82), P=1.23e-02 | - | - | M:0.33, E:0, P:0 | 5 |
| PREPL | NA | 1.37 (1.03-1.82), P=2.79e-02 | - | - | M:0.15, E:0, P:0 | 5 |
| SDHA | Energy Metabolism | - | 0.16 (0.04-0.58), P=5.82e-03 | - | M:0, E:0.4, P:0 | 5 |
| SDSL | NA | 2.09 (1.02-4.28), P=4.48e-02 | 0.58 (0.35-0.96), P=3.46e-02 | - | M:0.13, E:0.12, P:0 | 5 |
| SERHL2 | NA | 1.51 (0.95-2.4), P=2.21e-02 | - | - | M:0.18, E:0, P:0 | 5 |
| SLC25A1 | NA | 0.68 (0.5-0.92), P=1.31e-02 | - | - | M:0.32, E:0, P:0 | 5 |
| SLC25A22 | NA | 0.49 (0.25-0.97), P=3.94e-02 | - | - | M:0.11, E:0, P:0 | 5 |
| SLC25A37 | NA | 0.53 (0.3-0.95), P=2.65e-02 | - | - | M:0.12, E:0, P:0 | 5 |
| SLMO2 | NA | 0.92 (0.69-1.22), P=3.70e-02 | - | - | M:0.18, E:0, P:0 | 5 |
| SPG7 | NA | 0.61 (0.39-0.95), P=2.90e-02 | - | - | M:0.13, E:0, P:0 | 5 |
| STX17 | NA | 0.81 (0.69-0.96), P=1.36e-02 | - | - | M:0.31, E:0, P:0 | 5 |
| THG1L | NA | 0.55 (0.35-0.85), P=7.82e-03 | - | - | M:0.36, E:0, P:0 | 5 |
| TMEM177 | NA | 0.4 (0.19-0.87), P=2.12e-02 | 0.3 (0.11-0.81), P=1.82e-02 | - | M:0.29, E:0.21, P:0 | 5 |
| TMEM65 | NA | - | 0.16 (0.03-0.85), P=3.17e-02 | - | M:0, E:0.16, P:0 | 5 |
| TOP3A | NA | - | 0.43 (0.2-0.92), P=2.91e-02 | - | M:0, E:0.18, P:0 | 5 |
| TSPO | NA | 1.84 (1.1-3.07), P=2.01e-02 | - | - | M:0.26, E:0, P:0 | 5 |
| TST | Metabolic Process | - | 0.51 (0.33-0.78), P=2.06e-03 | - | M:0, E:0.56, P:0 | 5 |
| TSTD1 | NA | 0.58 (0.33-0.99), P=3.96e-02 | - | - | M:0.17, E:0, P:0 | 5 |
| TXNRD1 | NA | 0.84 (0.51-1.4), P=1.15e-02 | - | - | M:0.22, E:0, P:0 | 5 |
| UCP2 | NA | 0.64 (0.45-0.9), P=1.14e-02 | - | - | M:0.28, E:0, P:0 | 5 |
| UQCRFS1 | NA | 1.51 (1.13-2.02), P=5.11e-03 | - | - | M:0.35, E:0, P:0 | 5 |
| VPS13D | NA | 0.52 (0.29-0.95), P=3.34e-02 | - | - | M:0.11, E:0, P:0 | 5 |
