# Supplementary material for: Genetically prioritized mitochondrial regulators of advanced renal failure: multi-omic Mendelian randomization and biological plausibility assessment in allograft fibrosis
Source: Front Immunol. 2026 Mar 27;17:1783844. doi: 10.3389/fimmu.2026.1783844 (PMC13065693; doi:10.3389/fimmu.2026.1783844)
Supplement: Supplementary file 3 [file Table2.docx]

**Table S2. Primer sequences used for quantitative real-time PCR**

| **Gene** | **Species** | **Primer** | **Sequence (5′→3′)** | **Length (bp)** |
| --- | --- | --- | --- | --- |
| MRPS18C | Human | Forward | GTTTGCGGTGGTCTAGGGAG | 20 |
|  |  | Reverse | TGCTGGATACCTGTTGTGAAC | 21 |
| MRPS18C | Mouse | Forward | AACAGGTAACCAGCAATGAGG | 21 |
|  |  | Reverse | AGGGTCTTTGAGATATGCAGGAT | 23 |
| NDUFA13 | Human | Forward | GGCCCATCGACTACAAACGG | 20 |
|  |  | Reverse | CGCTCACGGTTCCACTTCATT | 21 |
| NDUFA13 | Mouse | Forward | ACGGCCCCATCGACTACAA | 19 |
|  |  | Reverse | CCTGGTTCCACCTCATCATTCT | 22 |
| C20orf72 | Human | Forward | CCCTTGTGGCTTTCTCTACTTC | 22 |
|  |  | Reverse | GGCAGCTTATGCTTGCTCAC | 20 |
| C20orf72 | Mouse | Forward | CAGTGGCTTCGTGTTCAGTG | 20 |
|  |  | Reverse | TGGTCAGCTTGTTGTCCTTG | 20 |
| MTIF3 | Human | Forward | CAAAAGCCTTTAGTACCGCTGA | 22 |
|  |  | Reverse | TGTTTCCCAAATCATTGCCCT | 21 |
| MTIF3 | Mouse | Forward | TACCTGACTTCTGCAAAAGGTTT | 23 |
|  |  | Reverse | CATAAGCTGGTACTCCGGTGG | 21 |
| β-Actin | Human | Forward | CATGTACGTTGCTATCCAGGC | 21 |
|  |  | Reverse | CTCCTTAATGTCACGCACGAT | 21 |
| β-Actin | Mouse | Forward | GGCTGTATTCCCCTCCATCG | 20 |
|  |  | Reverse | CCAGTTGGTAACAATGCCATGT | 22 |
| GAPDH | Human | Forward | GGAGCGAGATCCCTCCAAAAT | 21 |
|  |  | Reverse | GGCTGTTGTCATACTTCTCATGG | 23 |
| GAPDH | Mouse | Forward | AGGTCGGTGTGAACGGATTTG | 21 |
|  |  | Reverse | TGTAGACCATGTAGTTGAGGTCA | 23 |
