# Supplementary material for: Genetically prioritized mitochondrial regulators of advanced renal failure: multi-omic Mendelian randomization and biological plausibility assessment in allograft fibrosis
Source: Front Immunol. 2026 Mar 27;17:1783844. doi: 10.3389/fimmu.2026.1783844 (PMC13065693; doi:10.3389/fimmu.2026.1783844)
Supplement: Supplementary file 4 [file Table3.docx]

**Table S3. The antibodies used in this study**

| **Antibodies for western blotting** | **Company** | **Identifier** |
| --- | --- | --- |
| E-cadherin | Proteintech | 20874-1-AP |
| Fibronectin | Proteintech | 15613-1-AP |
| αSMA | Cell Signaling Technology | #19245 |
| TOMM20 | Proteintech | 66777-1-Ig |
| MRPS18C | ABclonal | A18453 |
| NDUFA13 (GRIM19) | Proteintech | 10986-1-AP |
| C20orf72 (MGME1) | Proteintech | 23178-1-AP |
| MTIF3 | Proteintech | 14219-1-AP |
| SOD2 | Santa Cruz | sc-137254 |
| β-ACTIN | Proteintech | 66009-1-Ig |
| Goat Anti-Mouse IgG | Proteintech | SA00001-1 |
| Goat Anti-Rabbit IgG | Proteintech | SA00001-2 |

| **Antibodies for immunofluorescence** | **Company** | **Identifier** |
| --- | --- | --- |
| E-cadherin | Proteintech | 20874-1-AP |
| MRPS18C | ABclonal | A18453 |
| NDUFA13 (GRIM19) | Proteintech | 10986-1-AP |
| C20orf72 (MGME1) | Proteintech | 23178-1-AP |
| MTIF3 | Proteintech | 14219-1-AP |
| SOD2 | Santa Cruz | sc-137254 |
| Cy™3 AffiniPure Goat Anti-Rabbit IgG | Jackson ImmunoResearch | 111-165-003 |
| Fluorescein (FITC) AffiniPure Goat Anti-Mouse IgG | Jackson ImmunoResearch | 115-095-003 |
| Fluorescein (FITC) AffiniPure Donkey Anti-Rabit IgG (H+L) | Jackson ImmunoResearch | 711-095-152 |
