# Supplementary material for: Genetically prioritized mitochondrial regulators of advanced renal failure: multi-omic Mendelian randomization and biological plausibility assessment in allograft fibrosis
Source: Front Immunol. 2026 Mar 27;17:1783844. doi: 10.3389/fimmu.2026.1783844 (PMC13065693; doi:10.3389/fimmu.2026.1783844)
Supplement: Supplementary file 5 [file Table4.docx]

**Supplementary Table 4. Instrument strength metrics for lead cis-QTL instruments used in SMR for the eight Tier 1 prioritized genes.**

| **Gene** | **Omics layer** | **Feature (probe/ID)** | **Lead SNP** | **QTL N** | **beta_QTL** | **SE_QTL** | **P_QTL** | **F-statistic** | **R²** |
| --- | --- | --- | --- | --- | --- | --- | --- | --- | --- |
| NDUFA13 | eQTL | ENSG00000186010 | rs11085264 (19:19621780 / G) | 22775 | 0.1876 | 0.01247 | 4.02×10^⁻51^ | 226.2 | 0.0098 |
| NDUFA13 | mQTL | cg08108768 | rs11085264 (19:19621780) | 1980 | -0.6953 | 0.04230 | <1.0×10^-300^ | 270.1 | 0.1202 |
| MRPS18C | eQTL | ENSG00000163319 | rs1565909 (4:84400330 / C) | 31349 | 0.1423 | 0.00799 | 5.93×10^⁻71^ | 317.2 | 0.0100 |
| MRPS18C | mQTL | cg14893161 | rs1565909 (4:84400330) | 1980 | -0.3946 | 0.03179 | <1.0×10^-300^ | 154.1 | 0.0723 |
| MTIF3 | mQTL | cg04384867 | rs9512686 (13:27992461) | 1980 | -0.4248 | 0.03374 | <1.0×10^-300^ | 158.5 | 0.0742 |
| C20orf72 (MGME1) | mQTL | cg14159672 | rs118056927 (20:33107241) | 1980 | 0.8947 | 0.14029 | 1.80×10^⁻10^ | 40.7 | 0.0201 |
| ECHDC1 | eQTL | ENSG00000093144 | rs9388571 (6:127706262 / T) | 30966 | 0.2832 | 0.00895 | 9.35×10^⁻220^ | 1001.1 | 0.0313 |
| ECHDC1 | mQTL | cg22536028 | rs9388571 (6:127706262) | 1980 | 0.7288 | 0.03539 | <1.0×10^-300^ | 424.0 | 0.1765 |
| MTHFD1L | eQTL | ENSG00000120254 | rs13201018 (6:151211007 / G) | 11496 | 0.5400 | 0.02970 | 7.99×10^⁻74^ | 330.5 | 0.0280 |
| MTHFD1L | mQTL | cg06809466 | rs13201018 (6:151211007) | 1980 | -0.8834 | 0.07157 | <1.0×10^-300^ | 152.3 | 0.0715 |
| QDPR | eQTL | ENSG00000151552 | rs10020773 (4:17526682 / C) | 31569 | -1.1016 | 0.00928 | <1.0×10^-300^ | 14094.4 | 0.3087 |
| QDPR | mQTL | cg18087406 | rs7661303 (4:17518920) | 1980 | 0.9553 | 0.03690 | <1.0×10^-300^ | 670.3 | 0.2531 |
| QDPR | pQTL | QDPR (Q09081) | rs7661303 (4:17518920) | 54219 | -0.4464 | 0.00705 | <1.0×10^-300^ | 4007.5 | 0.0688 |
| TST | eQTL | ENSG00000128311 | rs7290003 (22:37408829 / A) | 31171 | 0.2485 | 0.00956 | 5.82×10^⁻149^ | 675.5 | 0.0212 |

**Abbreviations:** eQTL, expression quantitative trait locus; mQTL, DNA methylation QTL; pQTL, protein QTL. Instrument strength was quantified as F-statistic = (β_QTL/SE_QTL)² (i.e., Z²). Variance explained was estimated as R² = F/(F + N - 2), where N denotes the effective QTL sample size reported for the specific SNP-feature association (and may vary across instruments). Data sources: eQTL from eQTLGen Phase I (Võsa et al., 2021); mQTL from McRae et al. (blood mQTL; N = 1,980; accessed via GoDMC mQTLdb); pQTL from Sun et al. (UKB-PPP; N = 54,219). GWAS outcome: kidney transplant status in UK Biobank (GCST90436425; N = 397,971). All instruments satisfy P_QTL < 5 × 10⁻⁸. Layers not shown for a given gene indicate that no genome-wide significant cis-QTL instrument was available in that layer under the selection criteria.
